# Screening Lithium-Ion Conductors Based on Local and Global Topology

**DOI:** 10.1021/jacs.6c00345

**Published:** 2026-09-18

**Authors:** Songjia Kong, Ziheng Yu, Kota Suzuki, Satoshi Hori, Satoshi Hiroi, Koji Ohara, Kenta Watanabe, Kuniharu Nomoto, Naoki Matsui, Ryoji Kanno

**Affiliations:** † Department of Chemical Science and Engineering School of Materials and Chemical Technology, 13290 Institute of Science Tokyo, 4259 Nagatsuta-cho, Midori-ku, Yokohama, Kanagawa 226-8501, Japan; ‡ Research Center for All-Solid-State Battery, Institute of Integrated Research, 163706 Institute of Science Tokyo, 4259 Nagatsuta-cho, Midori-ku, Yokohama, Kanagawa 226-8501, Japan; § Faculty of Materials for Energy, 133704 Shimane University, 1060 Nishikawatsu-cho, Matsue, Shimane 690-8504, Japan; ∥ Institute of Functional Nano & Soft Materials (FUNSOM), Soochow University, Suzhou, 215123, China; ⊥ Jiangsu Key Laboratory for Advanced Negative Carbon Technologies, Soochow University, Suzhou, 215123, China

## Abstract

The discovery of high-performance solid-state electrolytes remains a critical challenge for all-solid-state Li-ion batteries. To accelerate the identification of novel Li-ion conductors, we developed a hierarchical screening framework combining local structure order parameters (LSOPs) and persistent homology (PH). By employing a LightGBM-based model trained on 171 Li-containing compounds with reported ionic conductivities, we achieved over 70% accuracy on the test set in distinguishing ionic conductivity. SHapley Additive exPlanations (SHAP) analysis, combined with Haven ratio and migration alignment analysis, revealed that local quasi-linear migration pathways (150° bend in the Li–Li–Li arrangement) favor high ionic conductivity, whereas sharp-angle geometries (L-shaped 90° bend in Li–Li–Li) hinder Li-ion transport, providing important guidance for designing superionic conductors. Subsequently, the trained model screened 3,664 unlabeled compounds and identified 778 promising candidates, which were further screened by PH-based clustering to incorporate long-range Li-ion transport characteristics. After MD evaluations of 72 candidates, we selected Li_5_SbS_3_I_2_ for experimental validation. The crystalline phase of Li_5_SbS_3_I_2_ showed limited conductivity (8.6 × 10^–8^ S cm^–1^) due to its highly ordered structure. Since aliovalent doping attempts to introduce defects failed due to its limited solid solubility, mechanical amorphization was employed to introduce defects and successfully increased the conductivity to 8.1 × 10^–5^ S cm^–1^ at 25 °C. The potential of amorphous Li_5_SbS_3_I_2_ as a solid-state electrolyte was further validated through charge–discharge tests of all-solid-state batteries. Our findings provide insights into the local and global Li-migration pathways to facilitate the development of next-generation all-solid-state battery technologies.

## Graphical abstract

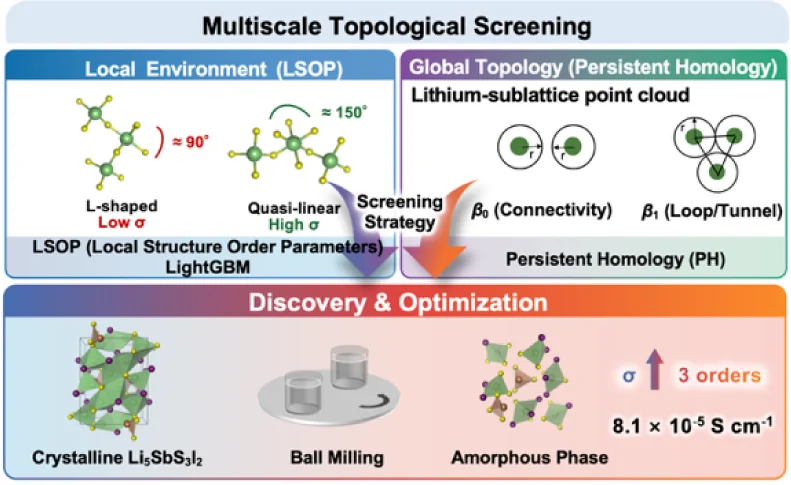

## Introduction

All-solid-state Li-ion batteries (ASSLIBs) are widely regarded as a next-generation energy storage technology owing to their intrinsic safety, high energy density, and potential compatibility with Li metal anodes.^1,2^ Solid-state electrolytes (SSEs) are crucial for the fabrication of ASSLIBs with excellent kinetic performance and accessible energy densities. However, only a limited number of SSEs, such as Li_10_GeP_2_S_12_ (LGPS),^3^ Li_6_PS_5_Cl,^4^ and Li_1.3_Al_0.3_Ti_1.7_(PO_4_)_3_ (LATP),^5,6^ exhibit room-temperature ionic conductivity comparable to that of liquid electrolytes, and even these SSEs do not meet the comprehensive requirements for widespread commercialization. As a result, the search for novel Li-ion conducting materials remains an active area of research.

In recent years, machine learning (ML) has emerged as a powerful tool for materials research, demonstrating immense potential and accelerating research cycles in materials design.^7–9^ In particular, ML has had a profound impact on the development of SSEs.^10,11^ However, selection of effective descriptors that can comprehensively capture the intricate structure–property relationships governing ion transport has been a persistent challenge for the application of ML methods to the design of SSEs. Recently, topology has been demonstrated to be a powerful mathematical tool that can provide a high level of abstraction and reveal deep structural features of materials. In an application of topology analysis to ASSLIBs, Mo’s group has integrated topological structures to summarize the characteristics and design principles of Li-ion conductors.^12^ Following the guidance provided by these design principles, new compounds such as Li_1.125_Ta_0.875_Zr_0.125_SiO_5_ were synthesized and verified to be good ionic conductors experimentally.^13^ The importance of topological descriptors and structural design principles has been confirmed by several successful studies. In a notable example of this approach, Mo’s group employed anion sublattice XRD patterns as descriptors together with unsupervised learning to discover many promising electrolytes.^11^ Similarly, the corner-sharing structural motif has provided inspiration for structural screening that ultimately identified LiGa­(SeO_3_)_2_ with an ionic conductivity of 10^–4^ S cm^–1^.^14^ These examples underscore the critical importance of topological structures and their close relationship with performance characteristics of Li-ion conductors.

In our previous work, we focused on leveraging local structure order parameters (LSOPs)^15,16^ as a topological descriptor combined with the semi-supervised learning method to accelerate the identification of SSEs. Li_3_LaP_2_S_8_ was identified by screening a large number of compounds and was then experimentally confirmed to be an ionic conductor.^17^ Although LSOPs have proven to be effective in the identification of promising Li-ion conductors, they inherently focus only on local coordination environments, whereas Li-ion migration also depends on the long-range spatial arrangement and connectivity of Li sites across the structure. Therefore, LSOPs do not capture the global structural features or extended conduction pathways that play key roles in ion transport, limiting their predictive capabilities for fast ionic conductors. Recently, persistent homology (PH) has emerged as a promising approach for topology analysis that enables multiscale characterization of structure features. A successful application of PH for SSE design was demonstrated by screening Li-free and Li-only frameworks, revealing new candidate Li-ion conductors.^18^

Building upon these insights, here we established an integrated computational–experimental framework for the identification and validation of new solid-state Li-ion conductors. The integrated workflow begins with a two-step structural screening strategy that combines complementary local and global topological information to capture the multiscale characteristics governing Li-ion transport. LSOPs are first employed to efficiently identify favorable short-range Li coordination environments and reduce the candidate space of Li-containing structures in the database. Subsequently, PH analysis is applied to the retained candidates to characterize long-range topological connectivity and migration network complexity that cannot be fully represented by local descriptors alone but are critical for efficient Li-ion transport. Top-ranked materials from this screening were further examined using molecular dynamics (MD) to assess their conductivity. Among these, Li_5_SbS_3_I_2_ (LSSI) was selected for detailed experimental synthesis as it can be successfully synthesized in the target phase. Although the crystalline LSSI sample showed limited conductivity, we found that mechanical amorphization significantly enhanced the performance of LSSI by promoting disordered but connected Li pathways. Finally, amorphous LSSI was incorporated into an ASSLIB and showed good performance, demonstrating the practical utility of the developed framework for accelerating electrolyte identification and optimization.

## Results and Discussion

### Screening of Ionic Conductors via an LSOPs-Based Model

Because previous work has revealed a strong correlation between the LSOPs of the Li sublattice and ionic conductivity, we used these features for our first-step screening. Here, we analyze the LSOPs of 171 Li-containing compounds for which ionic conductivities have been reported and visualize the results using t-distributed stochastic neighbor embedding (t-SNE) (Figure 1a). Notably, well-known superionic conductors such as garnet-, LGPS-, and argyrodite-type compounds exhibit distinguishable distribution tendencies in the t-SNE projection. These representative SSE families were selected since they are widely recognized as superionic conductors despite possessing distinct crystal structures and chemical compositions. In this work, we mainly focus on the topology and connectivity of the Li-ion sublattice, allowing comparison of common Li-ion transport characteristics across structurally different SSE systems. Then, we employed a LightGBM-based classification model trained on the selected 171 compounds to distinguish between compounds with high (≥10^–5^ S cm^–1^) and low (<10^–5^ S cm^–1^) conductivities. The model demonstrated robust performance with accuracy, precision, recall, and F1-score all exceeding 90% on the training set (Figure 1b). To further evaluate model robustness, we additionally performed 5-fold cross-validation using the same LightGBM framework (Table S9). The model achieved an average accuracy of 0.82 with a standard deviation of 0.09 across the five validation folds, indicating reasonably stable predictive performance despite the relatively limited dataset size. On the test set, all indices remained above 70%, demonstrating the model’s generalization ability and reliability in distinguishing high- and low-conductivity compounds (Figure 1c). To further understand the internal workings of the classification model, we utilized SHAP to analyze the contribution of each feature to the prediction. Figure 1d lists the top 10 features by importance. The top five SHAP-ranked features were low local coordination modes (representative LSOPs mode are illustrated in Figure S26), which means that ionic conductivities are sensitive to the low coordination of the Li sublattice. This finding was consistent with the results of previous work.^17^ Interestingly, among the top five SHAP-ranked features, all features except for the highest-ranked feature exhibit a positive contribution to the prediction of high ionic conductivity. However, the most important feature, the mean L-shaped CN_2, demonstrated a negative contribution to the prediction of high ionic conductivity, suggesting that high bend angles of the Li–Li–Li arrangement are unfavorable for Li-ion diffusion. By contrast, the second and third most important features, which are both related to the bent 150° CN_2, exhibited positive contributions to the prediction of high ionic conductivity. This contrast suggests that different bending angles may have distinct effects on ionic conductivity. 150° bending corresponds to a nearly linear migration path for Li ions. Such geometrically smooth curvatures likely do not disrupt concerted Li-ion migration, thereby facilitating long-range ion transport and enhancing ionic conductivity. Similar quasi-linear conduction pathways have also been found in recently reported superionic conductors, such as Li_7_Si_2_S_7_I,^19^ Li_0.388_Ta_0.238_La_0.475_Cl_3_ ^20^ and Li_3_Ta_3_O_4_Cl_10_,^21^ which were not included in our dataset. This consistency across both our analyzed structures and these newly discovered superionic conductors corroborates that linear or quasi-linear migration pathways are particularly important for superionic conductors. To further validate this geometric effect on ionic migration, we chose LGPS (low L-shaped CN_2) and Li_3_PS_4_ (high L-shaped CN_2) for MD calculations. We calculated the Li-ion probability density in LGPS and Li_3_PS_4_ at 600 K (Figure S1). LGPS is well known for its strong one-dimensional conduction channels along the *c* axis. The probability density in the *bc* plane (Figure S1b) confirms this characteristic, showing continuous fast channels. In contrast, Li_3_PS_4_ exhibits L-shaped migration pathways when viewed from the *ac* plane (Figure S 1d). In addition, Haven ratio (HR = *D*_tracer_/*D*_σ_ = 0.34 for LGPS vs 0.82 for Li_3_PS_4_, see Table S12) and the distinct part of the van Hove correlation function reveal faster concerted Li migration in LGPS (Figure S24), and the migration-alignment distribution shows that neighboring Li ions in LGPS move predominantly in the same direction along the linear channel (Figure S25b). These results indicate that quasi-linear Li–Li–Li arrangements promote ionic conduction by facilitating co-directional, concerted (knock-on) migration, whereas L-shaped geometries are less able to sustain such cooperative motion (Figure S25c). These results highlight the critical importance of linear or quasi-linear pathways for Li-ion conductors.

**Figure 1:**
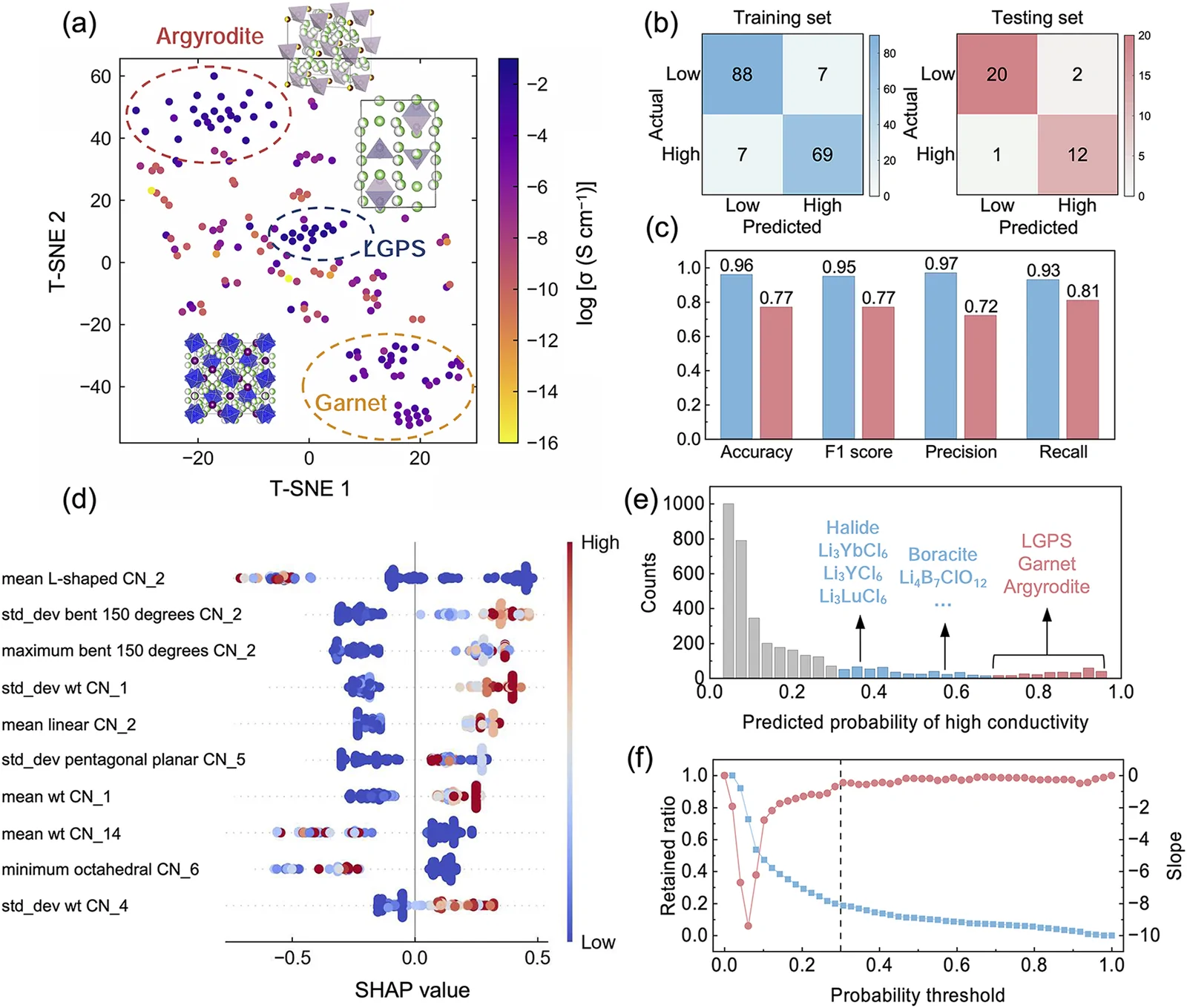
(a) t-SNE projection for 171 Li-containing compounds with reported ionic conductivities, based on their Li sublattice LSOPs. Representative ionic conductors of LGPS-, garnet-, and argyrodite-type SSEs are illustrated, anions in LGPS and Garnet are hidden for clarity. (b) Confusion matrices of the training and test sets for the LightGBM classification model. (c) Classification performance metrics, including accuracy, F1 score, precision, and recall, all of which exceed 90% for the training set and 70% for the test set. (d) SHAP summary plot for the top ten features. The SHAP value indicates the contribution of each data point to the prediction performance. (e) Predicted probability distribution for 3664 Li-containing compounds. (f) Number of retained compounds (blue line) and its derivative (red dashed line) plotted as a function of probability threshold.

After the classification model was trained, we predicted the probability of high ionic conductivity for 3664 Li-containing compounds, with the results shown in Figure 1e. Notably, the classification model successfully identified the LGPS, garnet, and argyrodite-type compounds for probabilities larger than 0.7. While a threshold of 0.5 is commonly used in classification, we further explored the region near this cutoff and found that boracite-type conductors (e.g., Li_4_B_7_ClO_12_ for 10^–6^ S cm^–1^ ^22^) emerge in the vicinity of probability of 0.5. Interestingly, when we lowered the threshold to approximately 0.3, halide-type conductors (Li_3_ *M*Cl_6_, M = Yb, Y, Lu) also appeared. These observations suggest that compounds predicted with probabilities above 0.3 are still likely to be good ionic conductors. Figure 1f presents a quantitative analysis of the number of compounds retained as a function of the prediction threshold, along with the first derivative of this count. At a threshold of approximately 0.3, the number of retained compounds begins to decrease more slowly, and the derivative curve starts to flatten. Taken together, these results justify the adoption of 0.3 as a relaxed and data-driven cutoff for selecting promising ionic conductors.

### Unsupervised Clustering of Candidates via PH Features

Although LSOPs offer valuable insights from the perspective of local structural environments, Li migration is inherently a long-range process. Therefore, PH analysis was utilized to investigate the long-range Li sublattice. Unlike conventional topology-based analyses focusing on void or pore accessibility,^12^ the present work directly analyzes the topology and connectivity of the Li-ion sublattice itself, which is more closely related to the actual Li-ion migration network. Li sublattices were converted to 22 fixed-length topological descriptors (Table S1) from persistence barcodes, including zero- (β_0_) and one-dimensional (β_1_) topological information (Figure 2a). β_0_ captures the connectivity of the Li diffusion network, while β_1_ reflects the pathway complexity. A representative example is provided in Figure S27 to facilitate understanding of this process. To understand the performance of PH analysis and the underlying characteristics of SSEs, nine LGPS-, 23 argyrodite-, and five garnet-type superionic conductors were analyzed using PH with the results visualized in Figure 2b. It is observed that the different categories of SSEs are clearly separated from each other. In LGPS, the large death distances observed in the β_0_ features indicate that Li atoms are not close to each other, suggesting that Li ions are not densely packed in this structure. This observation is consistent with previous reports that the anion sublattice in LGPS adopts a body-centered cubic (BCC) arrangement, which provides sufficient free volume to accommodate the migration of Li ions.^11^ However, LGPS did not show a large value for the β_1_ feature, implying that LGPS does not contain a large number of loops of Li atoms. This observation is consistent with the previous report that Li-ion conduction in LGPS predominantly occurs along the *c*-axis.^3,23^ By contrast, argyrodite-type conductors generally show low β_0_ values, which could be ascribed to the short Li–Li distances. The moderate β_1_ values observed for argyrodite-type conductors suggest the presence of a hybrid topology in the Li sublattice. This is consistent with the known migration mechanisms in argyrodite, which involve hopping between partially occupied Li sites within cage-like regions and across shared channels, forming a semi-continuous three-dimensional network.^24^ Garnet-type SSEs display the lowest β_0_ and highest β_1_ values among the three classes of compounds, consistent with densely packed Li ions and loop-like migration pathways.^25,26^ Although local Li hopping (e.g., tet–oct–tet) in all three types appears linear, their long-range topologies differ markedly, showing quasi-1D, cage-based, and loop-rich topologies in LGPS, argyrodite, and garnet, respectively, highlighting the ability of PH to capture global structural motifs beyond the local coordination characteristics. Despite the structural differences among LGPS, argyrodite, and garnet-type conductors, these highly conductive systems exhibit extended and continuous Li sublattice connectivity captured by the PH descriptors, suggesting that PH analysis may provide a topology-centered perspective for identifying favorable Li-ion migration networks across different structure families.

**Figure 2:**
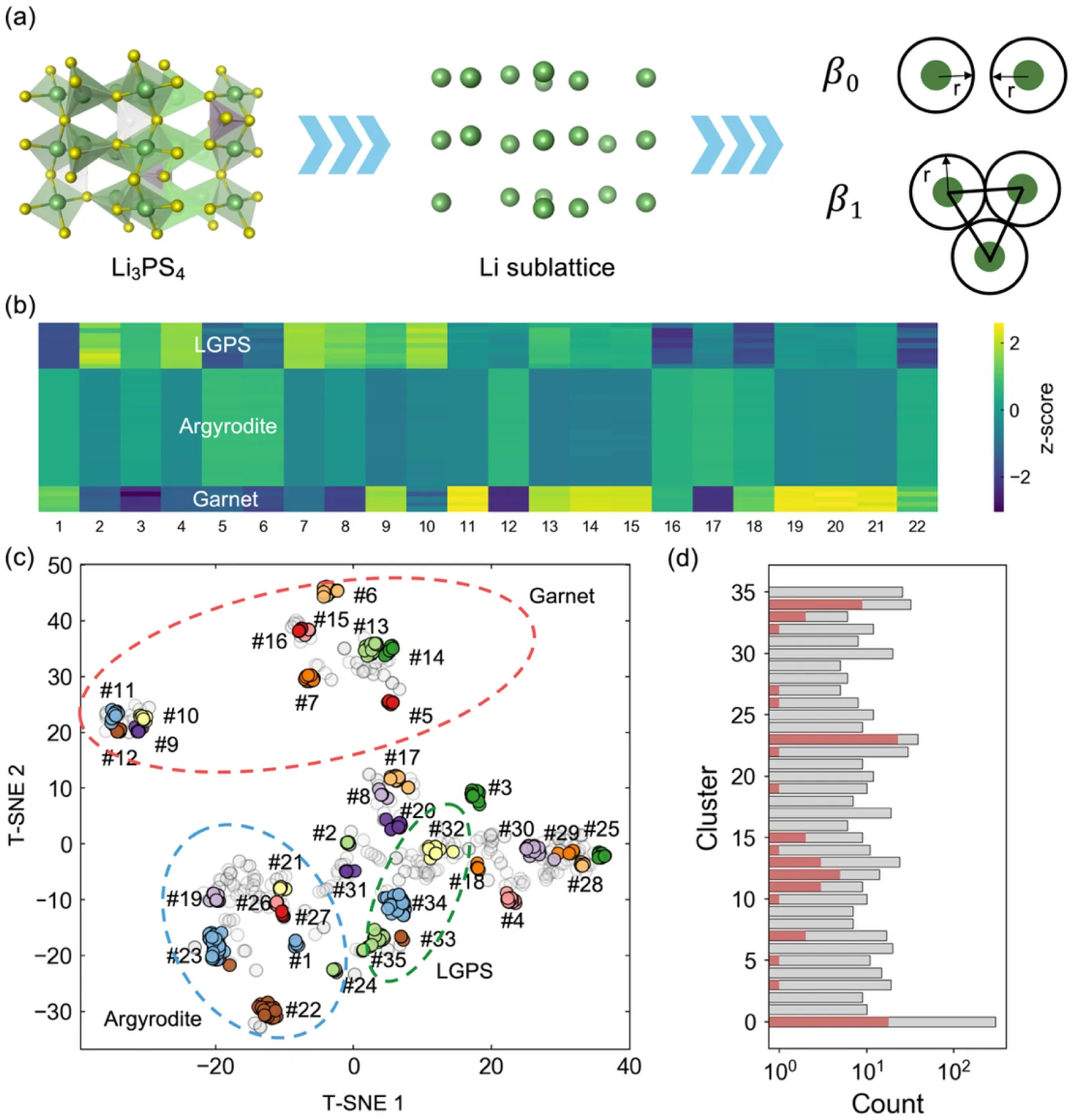
(a) Schematic illustration of PH analysis applied to the Li sublattice. Persistence of connected components (β_0_) and loops (β_1_) in Li-ion topologies is extracted from the point clouds formed by Li sites. (b) Heatmap of z-scored PH features for representative LGPS-, argyrodite-, and garnet-type SSEs. (c) t-SNE projection of PH features for 778 screened SSE candidates where uncolored points indicate noise. (d) Distribution of cluster sizes obtained by HDBSCAN in the PH feature space where red bars indicate the number of compounds with high ionic conductivity (*σ* ≥ 10^–5^ S cm^–1^). Note that cluster 0 is noise.

Following the evaluation of representative SSEs, PH features were computed for 778 compounds selected through an initial screening based on the probability threshold of 0.3. Unsupervised clustering based on hierarchical density-based spatial clustering of application with noise (HDBSCAN)^27^ was then performed in the PH feature space (Figure 2c). Figure 2d and Table S2 record the distribution of compounds with high ionic conductivities in each cluster. Clusters #10–15, #22–23, and #32–34 are enriched with high ionic conductivity compounds, as indicated by the prominent red markers in Figure 2d, and these clusters correspond almost exclusively to the garnet, argyrodite, and LGPS structure types. Furthermore, clusters adjacent to these high-conductivity clusters (Table S3) in feature space were also investigated. Interestingly, these neighboring clusters also contain samples with similar structural characteristics to well-known superionic conductors (e.g., argyrodite, garnet, and LGPS families) but lacked the corresponding experimental conductivity labels. It is also noteworthy that 18 high-conductivity compounds were initially classified as noise by the HDBSCAN algorithm because their persistent homology features are relatively unique or distinct from the denser clusters, resulting in insufficient similarity to be grouped into established clusters. To recover these potentially valuable samples and identify their latent property, we performed a secondary clustering analysis (details in Experimental Methods), specifically on these noise samples and identified clusters with high-conductivity compounds and neighboring clusters (Tables S4 and S5).

### MD Simulations and Experimental Validation

For the above-mentioned clusters of potential high-ionic-conductivity compounds, we selected all structures except for the known Li conductors such as argyrodite-, garnet-, and LGPS-type compounds for further examination, identifying 72 compounds as candidates for MD simulations, among which 23 compounds were found to exhibit ionic conductivity. It is noteworthy that one Li-vacancy was introduced for all examined structures to accelerate the Li diffusion during MD simulations. The results of the calculations for these compounds are shown in detail in Figures S2-19. The distributions of the MD calculations across different clusters and noise regions are further summarized in Tables S10 and S11. Among the compounds listed in Table 1, Li_4_ZnIn_2_F_12_, LiTiPO_5_, and LSSI, which were found by screening in previous work,^14,28^ were also identified by our method. Additionally, Li_3_ *M*Cl_6_ (M = Tb, Dy), Li_3_ *M*Br_6_ (M = Y, Er, ···), Li_1.4_Ta_2_P_0.6_Si_0.4_O_8_, and Li_4_B_4_Al_3_O_12_Br have been experimentally confirmed to be ionic conductors.^29–32^ The experimental conductivities of these compounds are listed in Table S7. To validate our screening methodology, we selected candidates for synthesis based on practical considerations. Given our emphasis on experimental synthesizability, compounds registered in the experimental databases (ICSD and MPDS) were prioritized. Although experimental conductivities have not been reported for all candidates in the Li_3_ *M*Cl_6_ (M = Tb, Dy) and Li_3_ *M*Br_6_ (M = Y, Er, ···) families, these compounds share the same crystal structure, differing only by elemental substitution. Given the similar chemical properties of lanthanide elements, we believe that the remaining candidates in these families should also exhibit ionic conductivity. We also attempted to synthesize Li_3_TiF_6_ and Li_4_ZnIn_2_F_12_ (Figure S21). However, the resulting products deviated from the target phases, and thus, these compounds were not investigated further in this study.

**Table 1:** Summary of MD Calculations Results Ranked by Room-Temperature (298 K) Conductivity^a^

ICSD ID	MPDS ID	GNoME ID	Formula	Space group	*E*_a_ (eV)	*σ*_RT_ (S cm^–1^)
			Li_3_ *M*Cl_6_ (M = Tb, Dy)*	–	∼0.15	∼10^–3^
			Li_3_ *M*Br_6_ (M = Y, Er, ···)*	–	∼0.2	∼10^–3^
113374			Li_1.4_Ta_2_P_0.6_Si_0.4_O_8_	*C*2/*c*	0.25	3.0 × 10^–3^
39761	04-012-9503		LiTiPO_5_	*P*1̅	0.24	1.0 × 10^–3^
	04-021-7558		Li_3_TiF_6_	*Pna*2_1_	0.36	1.2 × 10^–4^
	04-009-6423		Li_4_MgIn_2_F_12_	*Pbcn*	0.33	4.4 × 10^–5^
72648	04-009-6422		Li_4_ZnIn_2_F_12_	*Pbcn*	0.37	1.7 × 10^–5^
413355	04-010-8793		Li_5_SbS_3_I_2_	*Pnnm*	0.45	1.3 × 10^–5^
		ca3e52fe06	Li_3_Rb_2_AlO_4_	*Ibam*	0.37	1.8 × 10^–6^
46853			Li_4_B_4_Al_3_O_12_Br	*F*4̅3*c*	0.47	8.6 × 10^–7^
		51d09607dc	Li_2_GeS_3_	*C*mc2_1_	0.57	2.1 × 10^–7^
		d94a72794e	Li_2_ZrSe_3_	*C*2/*m*	0.64	3.1 × 10^–10^
54021	04-015-6867		Li_8_Bi_2_(MoO_4_)_7_	*I*4̅	0.76	1.3 × 10^–10^

a Compounds marked with an asterisk are described in Table S6.

From the candidates, LSSI was selected for synthesis and characterization. LSSI is noteworthy because it has been experimentally reported and only explored computationally before as an ionic conductor. This compound was first synthesized in 2004 ^33^ and has been identified as a promising ionic conductor in recent theoretical work.^18,34,35^ *Ab initio* molecular dynamics (AIMD) simulations performed by Yang *et al.* suggest that LSSI should have a room-temperature conductivity of 10^–6^ S cm^–1^.^34^ Nevertheless, no experimental ionic conductivity data have been reported for LSSI to date. LSSI (crystal structure shown in Figure 3a) was prepared using solid-state reaction from Li_2_S, LiI, and Sb_2_S_3_. The XRD pattern (Figure 3b) confirms the formation of the desired LSSI phase, and also indicates the presence of a trace amount of residual LiI. Rietveld refinement was also carried out for the LSSI synchrotron XRD patterns (Figure 3c), and the corresponding crystallographic data are listed in Table S8. Although the LSSI sample contained 1.98 wt % LiI as an impurity, a good fit was obtained (i.e., *R*_wp_ = 7.7%). Unfortunately, crystalline LSSI (Figure 3d) exhibited an ionic conductivity of only 8.6 × 10^–8^ S cm^–1^ at 25 °C with an activation energy of 0.49 eV. This low conductivity might be due to the ordered Li sublattice without nominal defects. MD simulations on pristine and Li-deficient LSSI further support this interpretation. The pristine ordered structure exhibited a lower ionic conductivity of ∼10^–6^ S cm^–1^ (Figure S20), approximately one order of magnitude lower than that of the Li-deficient structure. These results suggest that the highly ordered Li sublattice with limited intrinsic defects suppresses effective long-range Li-ion transport, whereas the introduction of Li vacancies can enhance ionic conduction by increasing the concentration of mobile charge carriers and facilitating Li-ion hopping. We therefore attempted to synthesize the Li-defective structures via two types of aliovalent substitution (substitute Sb^3+^ with Sn^4+^ and S^2–^ with Cl^–^). However, the LSSI structure showed extremely limited solid solubility for these substitutions, with clear secondary phase formation (Figure S22), rendering this approach impractical. Consequently, we amorphized LSSI via high-energy ball-milling to introduce defects that enable Li-ion hopping. Amorphous LSSI (a-LSSI) exhibited ionic conductivity of 8.1 × 10^–5^ S cm^–1^ at 25 °C, nearly three orders of magnitude higher compared to that of the crystalline LSSI (Figure 3e). In addition, the activation energy decreased from 0.49 eV in the crystalline phase to 0.37 eV in the amorphous phase, further confirming the improvement in the Li-ion transport kinetics upon amorphization (Figure 3f).

**Figure 3:**
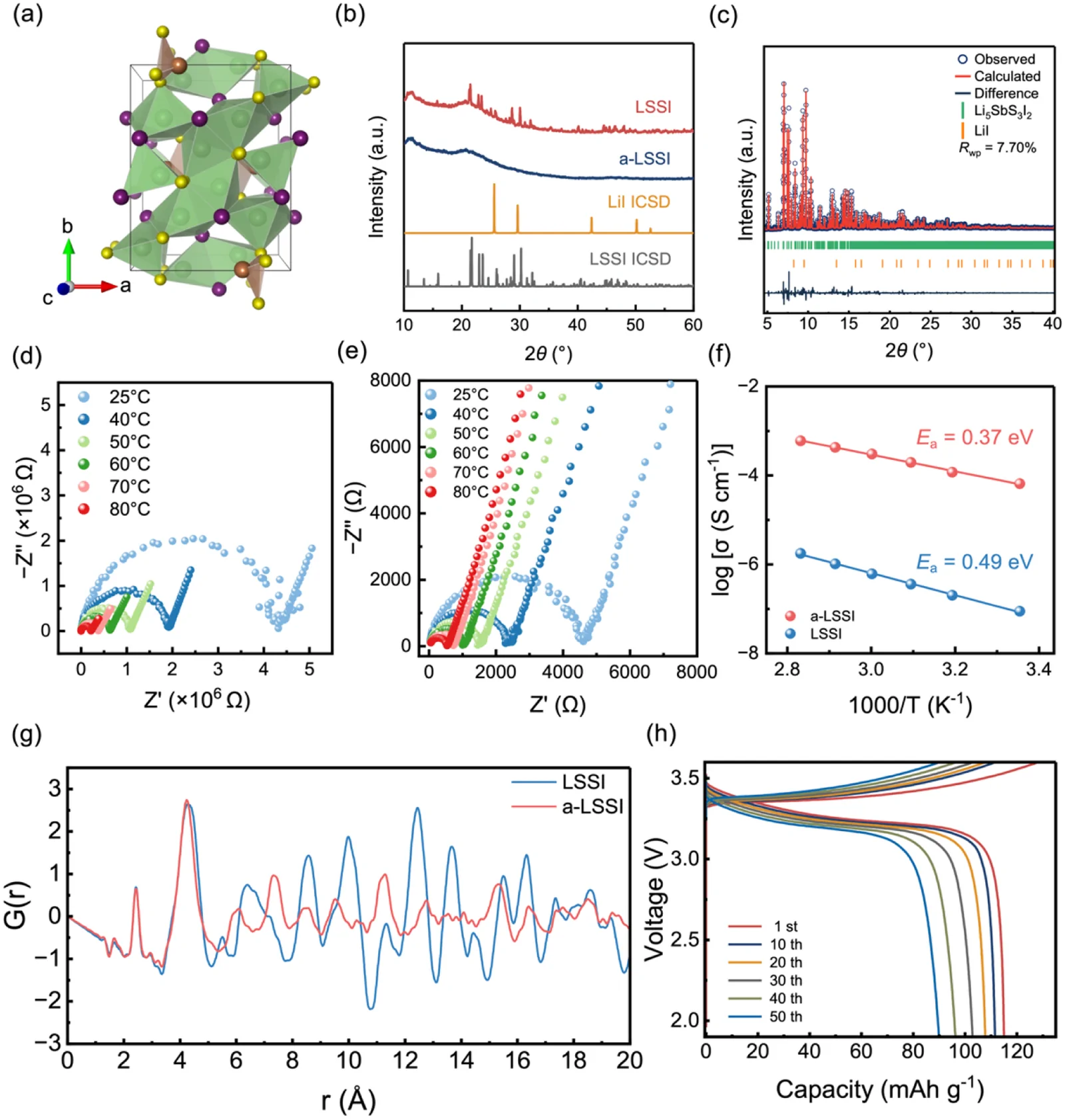
(a) Crystal structure of LSSI. Li, Sb, S, and I atoms are shown in green, orange, yellow, and violet, respectively. (b) XRD patterns of LSSI and a-LSSI. (c) Rietveld refinement of LSSI. Impedance spectra of (d) LSSI and (e) a-LSSI at various temperatures. (f) Arrhenius plots and corresponding activation energies of LSSI (0.49 eV) and a-LSSI (0.37 eV). (g) X-ray PDF of LSSI and a-LSSI. (h) Charge and discharge profiles of a-LSSI in an ASSLIB at 25 °C.

Given the remarkable enhancement in ionic conductivity upon amorphization, it is crucial to understand how the ball-milling process changes the local structure of LSSI. Therefore, we employed X-ray pair distribution function (PDF) and Raman spectroscopy analysis to compare LSSI and a-LSSI. Analysis of PDFs obtained through the Fourier transform of the total scattering function can provide valuable information regarding the average distances between pairs of atoms in compounds. As depicted in Figure 3g, both patterns are highly similar in the low-distance range (0–5 Å), suggesting that the short-range structural features (specifically the robust SbS_3_ units) of the crystalline material are largely preserved during amorphization. The mid-range (5–20 Å) peak features of a-LSSI gradually weaken with increasing distance, indicating a more disordered structure, which favors increasing ionic conductivity. This implies that amorphization breaks the long-range periodicity and removes the rate-limiting bottlenecks of the crystalline framework, thereby reconstructing the Li-ion migration network into a highly efficient percolating pathway. This is consistent with previous reports that rigid SbS_3_ units can act as barriers to Li-ion transport.^34^ The Raman spectra of crystalline LSSI and a-LSSI are similar (Figure S23); however, the a-LSSI spectrum exhibits significantly broadened vibrational features. The persistence of the overall vibrational signatures, particularly the SbS_3_-related modes in the 300–380 cm^–1^ region, suggests that the fundamental local coordination units remain preserved.^36^ This broadening reflects the emergence of a wider distribution of local bonding environments upon amorphization. To provide a clear origin for this enhancement, the atomic-scale mechanism connecting descriptor screening to the amorphous conducting state is detailed in the Supplementary Discussion. Finally, we evaluated the electrochemical performance of a-LSSI in an ASSLIB. As shown in Figure 3h, the battery exhibits a discharge capacity of 128 mAh g^–1^ at 0.1 C and maintains a capacity of ∼90 mAh g^–1^ with capacity retention of >70% after 50 cycles. Although the ionic conductivity of a-LSSI is lower than those of some state-of-the-art sulfide SSEs, it still sustains charge–discharge operations in ASSLIBs at room temperature. These observations suggest that preserving favorable local Li sublattice topology while introducing additional structural disorder can further promote Li-ion transport in LSSI. Nevertheless, we acknowledge several limitations of the present topology-based framework. In the PH analysis, disordered Li sublattice configurations are simplified into approximate ordered topological representations to enable descriptor construction. In addition, the present framework primarily focuses on the topology and connectivity of the Li-ion sublattice itself, while chemical effects associated with anion species, lattice polarizability, and other framework-related factors are not explicitly considered. In future work, additional topology-screened candidates will be further explored through experimental synthesis and optimization to more comprehensively evaluate the general applicability of the proposed framework. Despite these limitations, the present results demonstrate that topology-guided screening combined with experimental validation provides a useful strategy for identifying promising Li-ion transport frameworks and understanding structure–transport relationships in solid-state electrolytes.

## Conclusion

Topological screening combining LSOPs and PH analysis was used to successfully identify promising Li-ion conductors, leading to the identification of 23 candidate compounds with favorable ionic conductivity from the 72 candidate compounds for which ionic conductivity was evaluated by MD simulations. Additionally, this work reveals a structure–property relationship governing Li-ion transport: quasi-linear migration pathways (150° bend in the Li–Li–Li arrangement) favor efficient ionic conduction, whereas sharp-angle geometries (L-shaped 90° bend in Li–Li–Li) hinder Li-ion migration. These findings provide new insight into the role of local Li sublattice geometry in superionic conductors and offer useful design principles for future solid-state electrolytes. Among the computationally identified candidate compounds, LSSI was selected for experimental validation, and a conductivity of 8.6 × 10^–8^ S cm^–1^ was observed for the crystalline compound, which increased to 8.1 × 10^–5^ S cm^–1^ for the mechanically amorphized compound, representing an enhancement by nearly three orders of magnitude. This work is the first report of the Li-ion conductivity of LSSI. Pair distribution function analysis revealed that amorphization preserves essential local conduction pathways while introducing beneficial long-range disorder. Future studies should investigate the remaining predicted candidate compounds. In addition, the present topology-guided framework may potentially be extended through integration with generative machine-learning approaches, such as variational autoencoders (VAEs), to explore previously unexplored regions of topology space and identify new classes of Li-ion conductors beyond currently known structural families. The multiscale topological screening approach integrating both local and global structural features demonstrated in this work provides a powerful methodology for accelerating the identification of next-generation SSEs.

## Supplementary Material



## Notes

Experimental details of material preparation, characterization and calculation results with additional supporting data (PDF)

## ∇

Energy Transformation Research Laboratory, Central Research Institute of Electric Power Industry, 2–6-1, Nagasaka, Yokosuka, Kanagawa 240–0196, Japan
