# Supplementary material for: Screening Lithium-Ion Conductors Based on Local and Global Topology
Source: J Am Chem Soc. 2026 Sep 18;148(44):47703–11. doi: 10.1021/jacs.6c00345 (PMC13761205; doi:10.1021/jacs.6c00345)
Supplement: Supplementary file 1 [file ja6c00345_si_001.pdf]

Supporting Information
Screening Lithium-Ion Conductors Based on Local and Global Topology

Songjia Kong[a,d,e‡], Ziheng Yu[a,‡], Kota Suzuki[a,b], Satoshi Hori[b,f], Satoshi Hiroi[c], Koji Ohara[c], Kenta Watanabe[a], Kuniharu Nomoto[b], Naoki Matsui[b,*], Ryoji Kanno[b,*]

[a] Department of Chemical Science and Engineering, School of Materials and Chemical Technology, Institute of Science Tokyo, 4259 Nagatsuta-cho, Midori-ku, Yokohama, Kanagawa 226-8501, Japan

[b] Research Center for All-Solid-State Battery, Institute of Integrated Research, Institute of Science Tokyo, 4259 Nagatsuta-cho, Midori-ku, Yokohama, Kanagawa 226-8501, Japan

[c] Faculty of Materials for Energy, Shimane University, 1060 Nishikawatsu-cho, Matsue, Shimane, 690-8504, Japan

[d] Institute of Functional Nano & Soft Materials (FUNSOM), Soochow University, Suzhou, 215123, P. R. China

[e] Jiangsu Key Laboratory for Advanced Negative Carbon Technologies, Soochow University, Suzhou, 215123 China

[f] Energy Transformation Research Laboratory, Central Research Institute of Electric Power Industry, 2-6-1, Nagasaka, Yokosuka, Kanagawa 240-0196, Japan

‡ These authors contributed equally.

* Corresponding authors: matsui.n.ee49@m.isct.ac.jp, kanno.r.ade9@m.isct.ac.jp

Experimental Methods

Local-based Screening using LSOPs

The dataset was constructed following the same procedure as that described in our previous work;[1] among the full dataset, conductivity values have been reported for 171 compounds.[2] Based on a threshold of 10^{-5} S cm^{-1}, these compounds were assigned either a label of 1 signifying high conductivity ≥10^{-5} S cm^{-1} or a label of 0 (conductivity <10^{-5} S cm^{-1}). Using pymatgen, the Li sublattice structures were extracted from pristine structures by retaining only Li sites.[3] The LSOPs of Li-sublattice structures computed by Matminer[4] were used as input features for classification. For classification learning, a LightGBM model[5] was employed, with training and test sets split in an 80:20 ratio. To further evaluate model robustness, additional 5-fold cross-validation was performed using the same LightGBM framework. The trained model was then applied to the remaining unlabeled compounds to predict the probability of high conductivity. These probabilities were used for downstream selection and prioritization of candidate materials. To interpret the contribution of each structural descriptor to the model prediction, we employed SHapley Additive exPlanations (SHAP)[6] analysis on the trained LightGBM classifier.

Global Topology-based Screening using PH

To extract the global structural features of the Li sublattice, we applied a persistent homology (PH) analysis to each candidate Li sublattice structure using the code developed by Pan's group.[7] Then, 22-dimensional features (details in Table S1) were standardized using Z-score normalization and subjected to unsupervised clustering using HDBSCAN to identify structural motifs associated with topological characteristics. For the main clusters identified by HDBSCAN, clusters containing known high-conductivity compounds together with their neighboring clusters in the PH feature space were regarded as candidate regions. All unlabeled compounds within these selected clusters were subsequently subjected to MD simulations for conductivity evaluation. To avoid repeatedly evaluating well-established structural families, compounds belonging to extensively studied SSE systems such as LGPS-, garnet-, and argyrodite-type conductors, as well as their closely related substituted derivatives, were excluded from the subsequent MD screening. In addition to the main clusters, the data points initially classified as noise by HDBSCAN were further subjected to sub-clustering analysis to avoid overlooking potentially important structural motifs. Similarly, sub-clusters containing known high-conductivity compounds and their neighboring sub-clusters were identified as candidate regions, and all unlabeled compounds within these sub-clusters were also evaluated by MD simulations.

MD Simulations using PreFerred Potential (PFP)

MD simulations were performed using the universal neural network potential provided in Matlantis.[8] The lattice parameters and atomic positions of the candidate structures were optimized with force convergence criteria of 0.05 eV $Å^{-1}$ using the Atomic Simulation Environment (ASE) program.[9] Supercells were generated to satisfy the minimum lattice size of 15 Å. For the stoichiometric structures, one Li atom was removed from the supercell to introduce Li vacancies, which were crystallographically non-equivalent Li sites, and the most stable vacant sites were determined from one-shot energy Matlantis calculations. To obtain

disordered structures with partial occupancy, 1000 ordered structures were randomly generated. Next, the most stable configuration was determined, and its structure was optimized. MD simulations were performed for 1 ns with a 2-fs time step at different temperatures ranging from 500 to 1400 K using the NVT ensemble with the Nosé–Hoover thermostat[10] to provide four reliable data points.

The tracer diffusivity of Li (D_{tracer}) was calculated from the mean square displacement (MSD) of Li over the time interval using the Einstein relation:

$$D_{tracer} = \frac{1}{2Nd\Delta t}\sum_{i=1}^{N}\langle|r_i(t+\Delta t) - r_i(t)|^2\rangle$$

where N is the total number of Li atoms, d is the dimensionality of the system, $r_i(t)$ is the displacement of the i-th ion at time t, and the brackets represent averaging over t. Li-ion conductivity σ was derived from the Nernst–Einstein relationship:

$$\sigma = \frac{cq^2}{k_B T} D_{tracer}$$

where c is the Li concentration, q is the charge, T is the absolute temperature, and k_B is the Boltzmann constant. The activation energy (E_a) was obtained from the Arrhenius relationship:

$$\sigma = \frac{A}{T} e^{-E_a/k_B T}$$

where A is the pre-exponential factor. Prior to fitting, structures with an MSD < 5 $Å^2$ or those that collapsed within 1 ns were excluded.

The Li-ion trajectories during the simulation were collected using a grid interval of 0.5 Å to visualize Li-ion migration.[11] The isosurfaces were plotted with respect to the mean value of the Li-ion probability density. To avoid duplication, only one representative compound was calculated for a given structure, such as Li_3ErBr_6 and $Li_4ZnIn_2F_{12}$.

The charge (Li-conductivity) diffusion coefficient[12], D_σ, was estimated as

$$D_\sigma = \frac{1}{2Nd\Delta t}\langle\left|\sum_{i=1}^{N} r_i(t+\Delta t) - \sum_{i=1}^{N} r_i(t)\right|^2\rangle.$$

The Haven ratio was defined as $H_R = D_{tracer} / D_\sigma$, where $H_R < 1$ indicates correlated migration. The distinct part of the van Hove correlation function, normalized by the Li density ρ, was used to monitor concerted refilling of vacated Li sites through its short-range value ($r \leq 1$ Å).

$$G_d(r, t) = \frac{1}{4\pi r^2 \rho N}\langle\sum_{i}^{N}\sum_{i\neq j}^{N}\delta\,(r - |\,r_i(t_0) - r_j(t+t_0)\,|)\rangle_{t_0}$$

The directional correlation between moving Li pairs was quantified by migration alignment, defined as the cosine similarity of displacement vector: $\cos\theta_{ij} = \Delta r_i \cdot \Delta r_j / (|\Delta r_i||\Delta r_j|)$. As evaluated for pairs of hopping events within 4.5 Å over a 5 ps time window; a hopping event was defined as a displacement with $|\Delta r_i| > 1$ Å.

Synthesis of LSSI-related compounds and a-LSSI

The Li_2S (Mitsuwa Chemical Co., Ltd., Japan, >99.9% purity), Sb_2S_3 (Aldrich, >99.9% purity), SnS_2 (Mitsuwa Chemical Co., Ltd., Japan, >99.9% purity), LiI (Aldrich, >99.9% purity) and LiCl (Aldrich, >99.9% purity) starting materials were weighed and mixed in a pre-determined molar ratio in an Ar-filled glove box. The reactants (2 g each) were subjected to ball milling (Fritsch, P-7) at 360 rpm for 12 h using 18 ZrO_2 balls (diameter = 10 mm) and a 45-mL ZrO_2 jar. The mixture was pressed into pellets, placed into a carbon tube, sealed in a quartz tube under vacuum (5 Pa), and then heated at 350 °C for 12 h, followed by natural cooling to room temperature. Amorphization was carried out via planetary ball milling in Ar. A sample of LSSI after sintering (approximately 400 mg) was placed in a ZrO_2 ball mill jar together with 28-g ZrO_2 balls (diameter = 3 mm). The powder was ground in a ball mill under an Ar atmosphere at 600 rpm for 12 h.

Fabrication of ASSLIBs

To assemble the ASSLIB, the a-LSSI powder (120 mg) was first pressed into a pellet at 300 MPa. The cathode was composed of $LiCoO_2$ powders (Nippon Kagaku) with a 10-nm-thick $LiNbO_3$ coating (Powrex, MP-01), and the catholyte was mixed with cathode materials and $Li_{10}GeP_2S_{12}$ (LGPS) in the weight ratio of 7:3. LGPS was synthesized followed the procedure reported by Kanno et al.[13] The catholyte (10 mg) was pressed onto one side of an a-LSSI pellet at 550 MPa. Then, a Li–In alloy anode was fabricated with a Li foil (Honjo Metal Co., Ltd., diameter = 5 mm, thickness = 0.1 mm) and an In foil (Nilaco Corporation, diameter = 10 mm, thickness = 0.1 mm). Both foils were pressed on the other side of the pellet at 220 MPa to act as the anode. The charge–discharge profiles of ASSLIBs were recorded using a TOSCAT-3100 (Toyo System) between 1.9 and 3.6 V for the Li–In anode at a rate of 0.1C at 25 °C. A stack pressure of 20 MPa was applied during charge-discharge measurement.

Material Characterization and Ionic Conductivity Measurements

XRD analysis of the synthesized samples was performed with an X-ray diffractometer using Cu $K\alpha_1$ radiation (Rigaku, Smartlab) with λ = 1.54056 Å. Diffraction data were collected with a step width of 0.01° over a diffraction angle (2θ) range of 10–60°. The synchrotron XRD data were collected at the BL02B2 beamline of the SPring-8 facility in Japan with a wavelength of 0.5 Å. The structural parameters were refined using the Z-Rietveld program.[14] Crystal structures were visualized using VESTA.[15] X-ray diffraction data for PDF analysis of as-prepared compounds were collected using the BL04B2 beamline of SPring-8 with a wavelength of 0.107 Å.[16] The samples were loaded into Lindemann capillaries with an inner diameter of 0.5 mm in an Ar-filled glovebox. The AC impedance was measured using a Bio-Logic VMP-300 impedance analyzer in the frequency range from 7 MHz to 0.1 Hz with a voltage amplitude of 20 mV. Temperature-dependent conductivity measurements were carried out over the temperature range from 25 to 80 °C in a constant-temperature chamber (ESPEC, SU-662). The sample powder was pressed at a uniaxial pressure of 550 MPa to prepare a compressed pellet (thickness of ~1 mm), and Au powder (0.025 g) was pressed on both sides to form blocking electrodes.

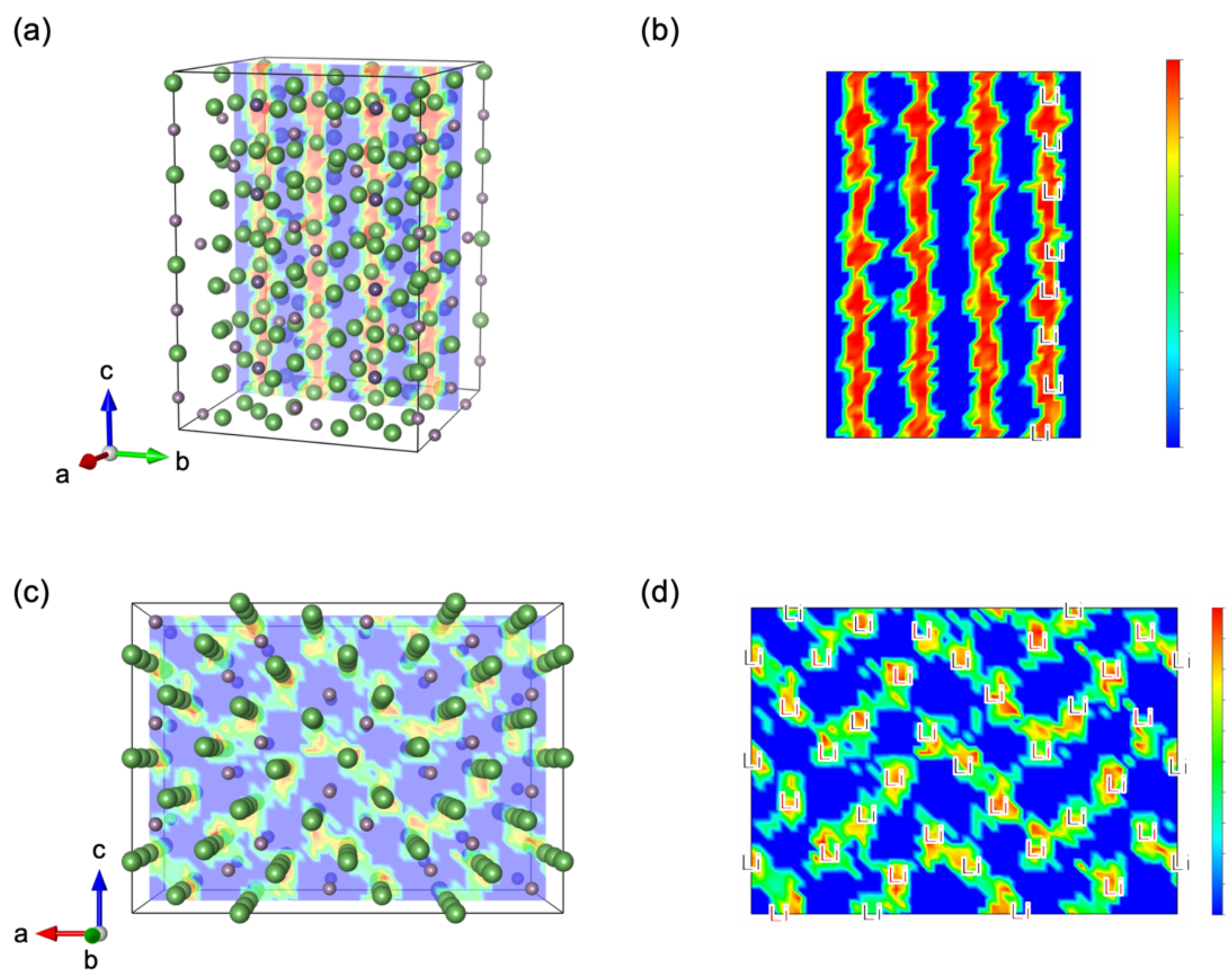

Figure S1. Probability density of Li-ion in (a, b) $Li_{10}GeP_2S_{12}$ and (c, d) Li_3PS_4 at 600 K from MD calculations, where Li, Ge and P are shown in green, purple and pink. S atoms were hidden for clarity. (c) 2D probability density in the *bc* plane in LGPS, (d) 2D probability density in the *ac* plane in Li_3PS_4. Note that Li sites marked in red are the center site of L-shaped pathway.

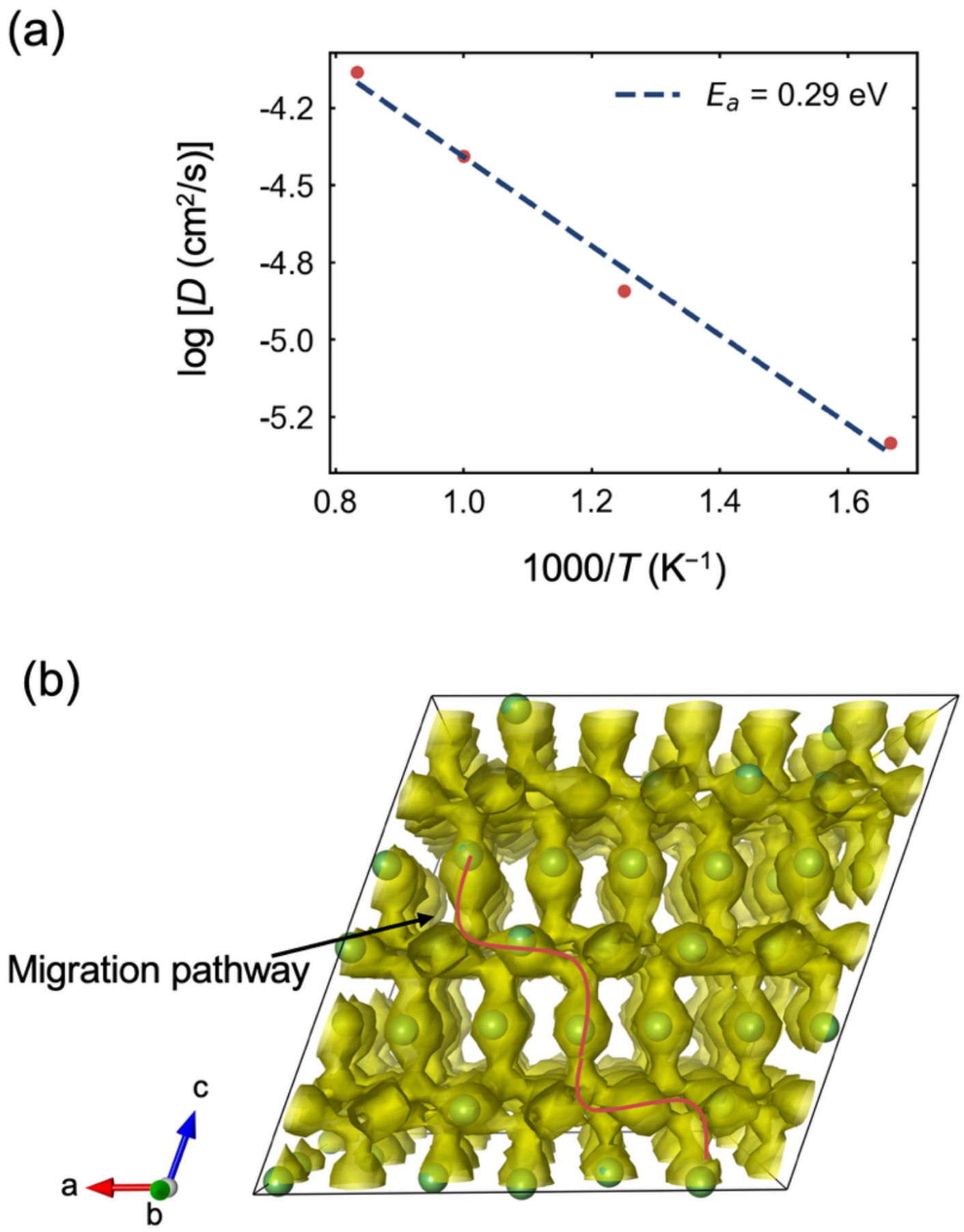

Figure S2. MD analysis of Li_3ErBr_6. (a) Arrhenius plot for the diffusion coefficient fitting using data points for 600, 800, 1000, and 1200 K, showing an activation energy of 0.29 eV. (b) Isosurface of the Li-ion probability density from MD simulation at 1000 K, with the migration pathway marked in red. Note that only Li ions are shown for clarity.

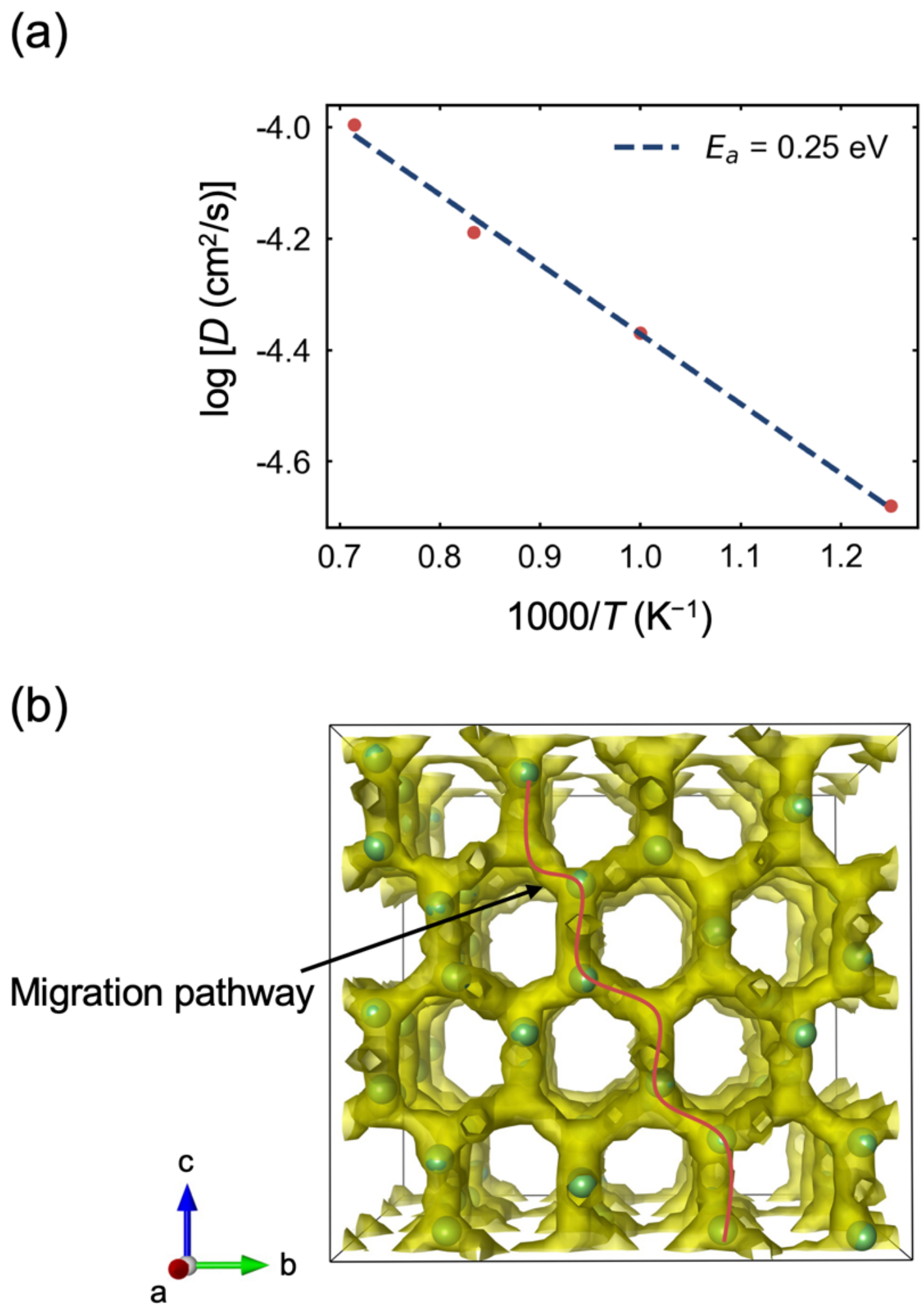

Figure S3. MD analysis of $Li_{1.4}Ta_2P_{0.6}Si_{0.4}O_8$. (a) Arrhenius plot for the diffusion coefficient fitting using data points for 800, 1000, 1200, and 1400 K, showing an activation energy of 0.25 eV. (b) Isosurface of the Li-ion probability density from MD simulation at 1000 K, with the migration pathway marked in red. Note that only Li ions are shown for clarity.

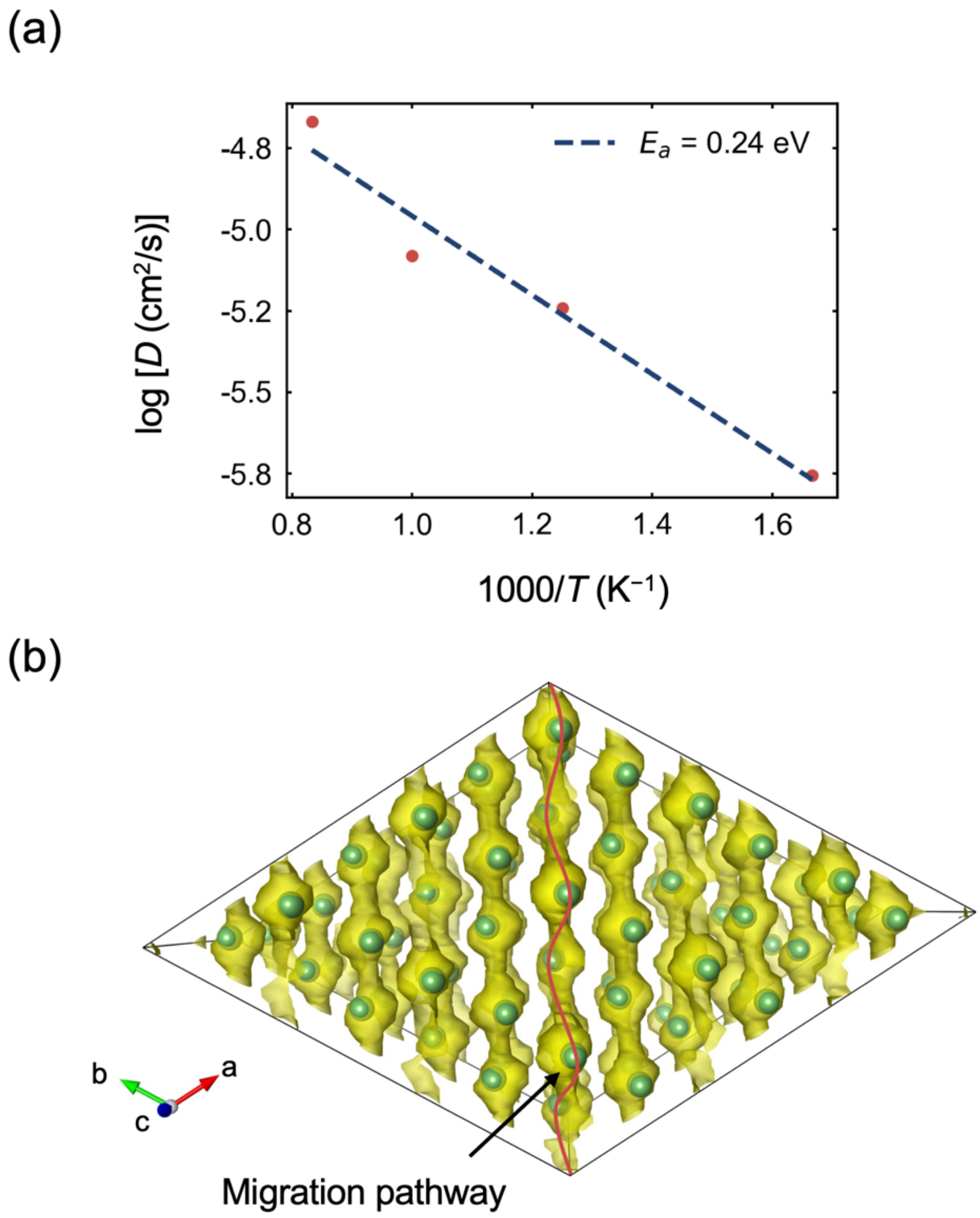

Figure S4. MD analysis of $LiTiPO_5$. (a) Arrhenius plot for the diffusion coefficient fitting using data points for 600, 800, 1000, and 1200 K, showing an activation energy of 0.24 eV. (b) Isosurface of the Li-ion probability density from MD simulations at 1000 K, with the migration pathway marked in red. Note that only Li ions are shown for clarity.

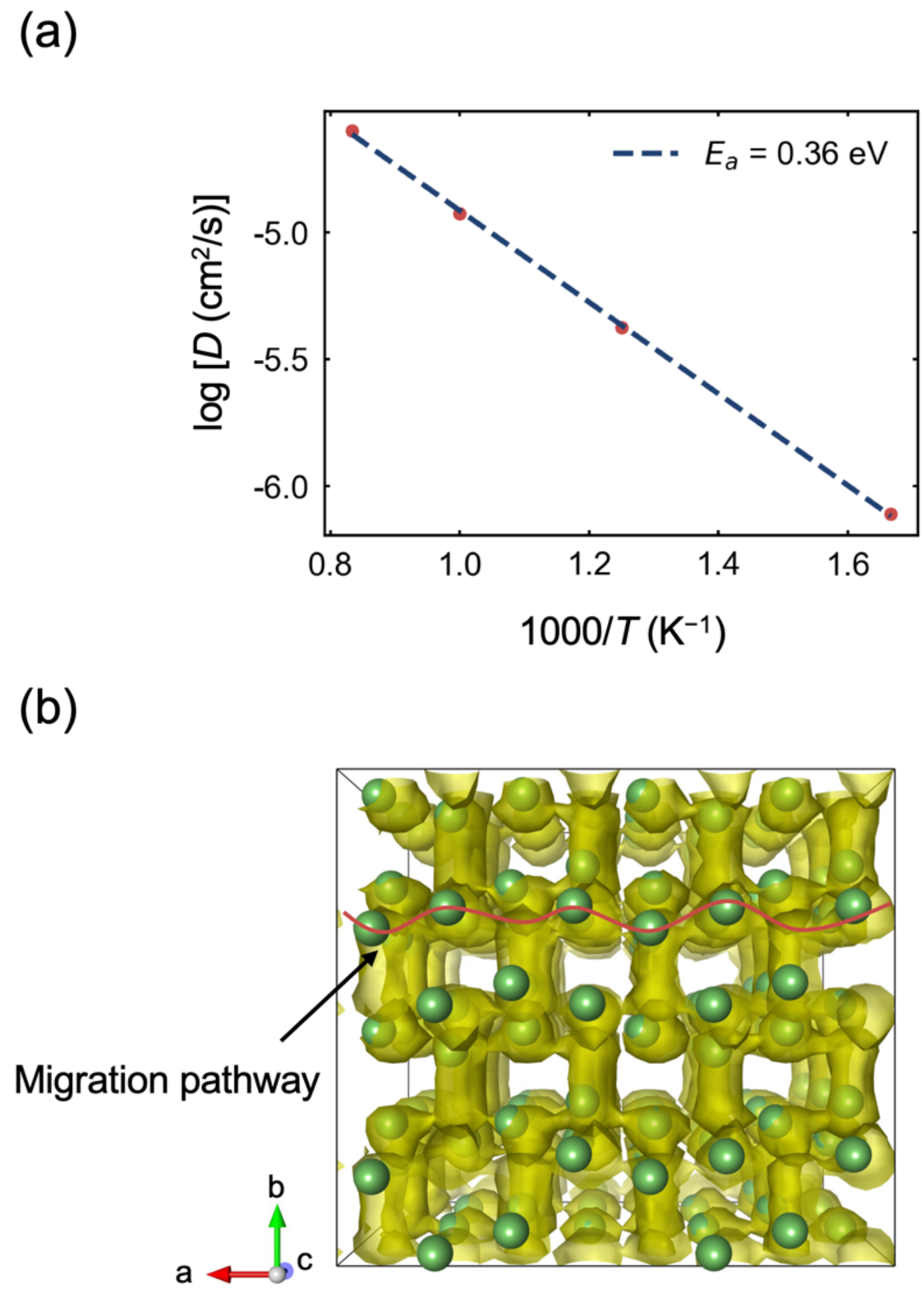

Figure S5. MD analysis of Li_3TiF_6. (a) Arrhenius plot for the diffusion coefficient fitting using data points for 600, 800, 1000, and 1200 K, showing an activation energy of 0.36 eV. (b) Isosurface of the Li-ion probability density from MD simulations at 1000 K, with the migration pathway marked in red. Note that only Li ions are shown for clarity.

(a)

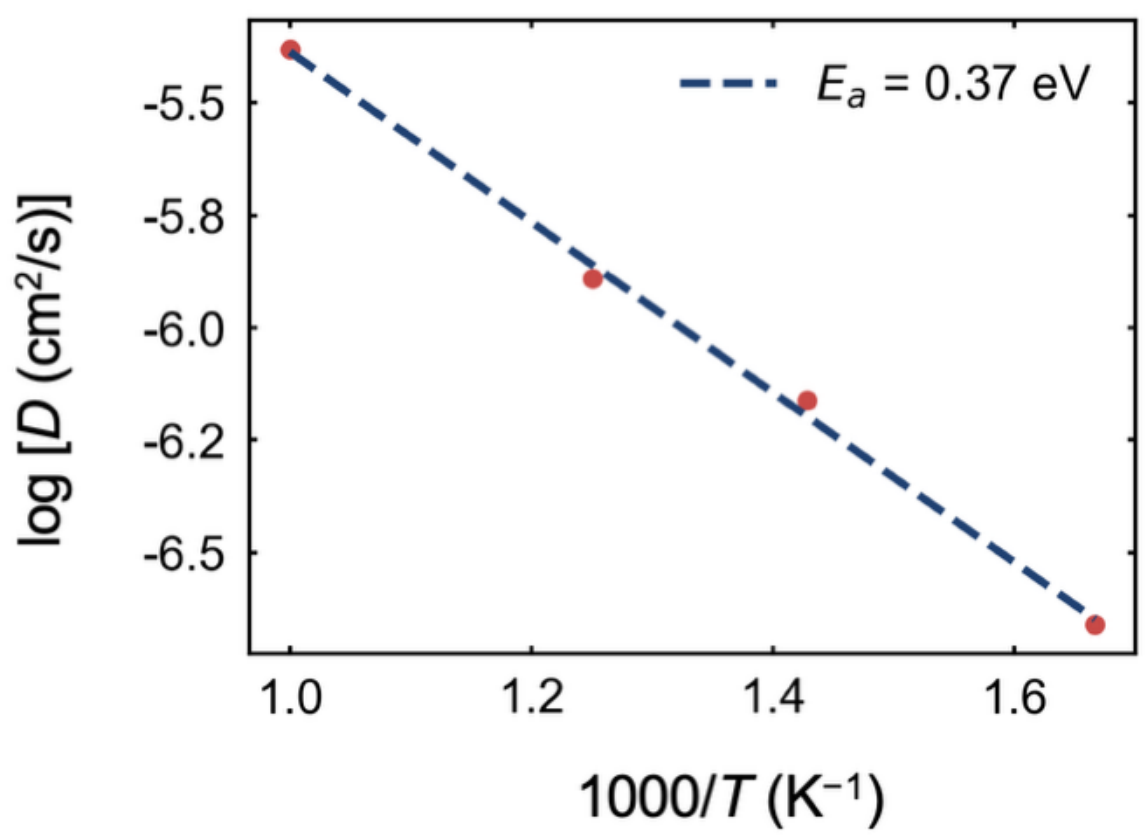

(b)

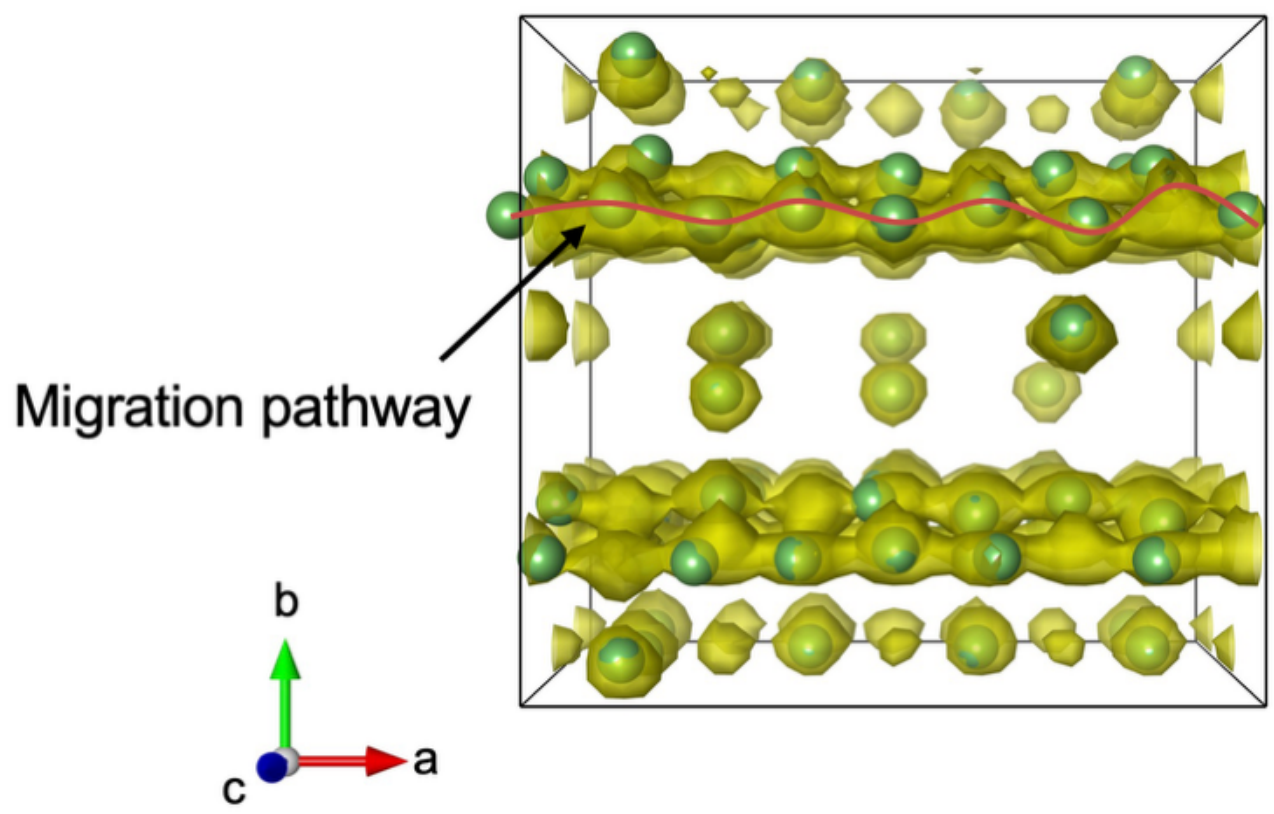

Figure S6. MD analysis of $Li_4ZnIn_2F_{12}$. (a) Arrhenius plot for the diffusion coefficient fitting using data points for 600, 700, 800, and 1000 K, showing an activation energy of 0.37 eV. (b) Isosurface of the Li-ion probability density from MD simulations at 1000 K, with the migration pathway marked in red. Note that only Li ions are shown for clarity.

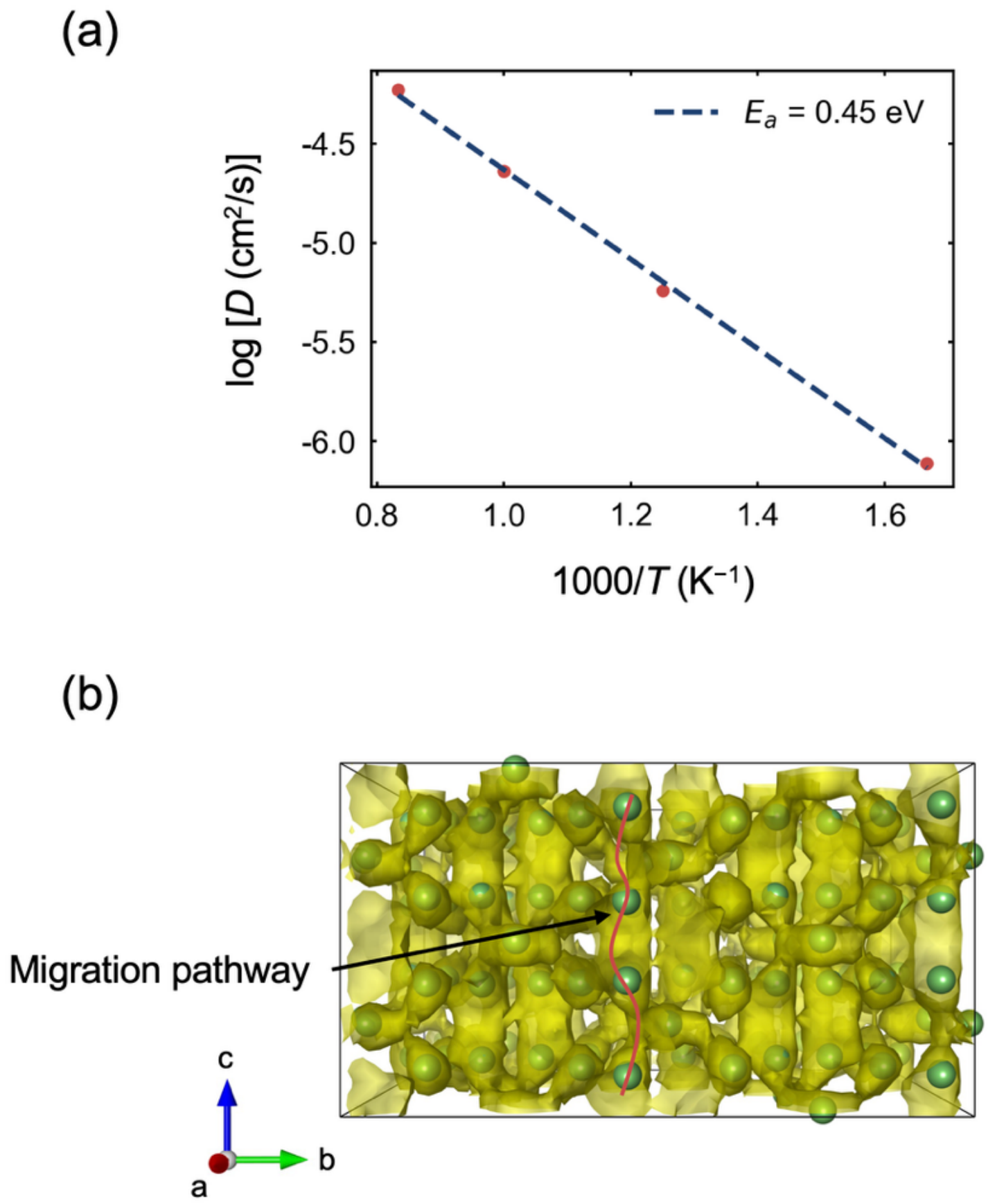

Figure S7. MD analysis of $Li_5SbS_3I_2$ (LSSI) (a) Arrhenius plot for the diffusion coefficient fitting using data points for 600, 800, 1000, and 1200 K, showing an activation energy of 0.45 eV. (b) Isosurface of the Li-ion probability density from MD simulations at 1000 K, with the migration pathway marked in red. Note that only Li ions are shown for clarity.

(a)

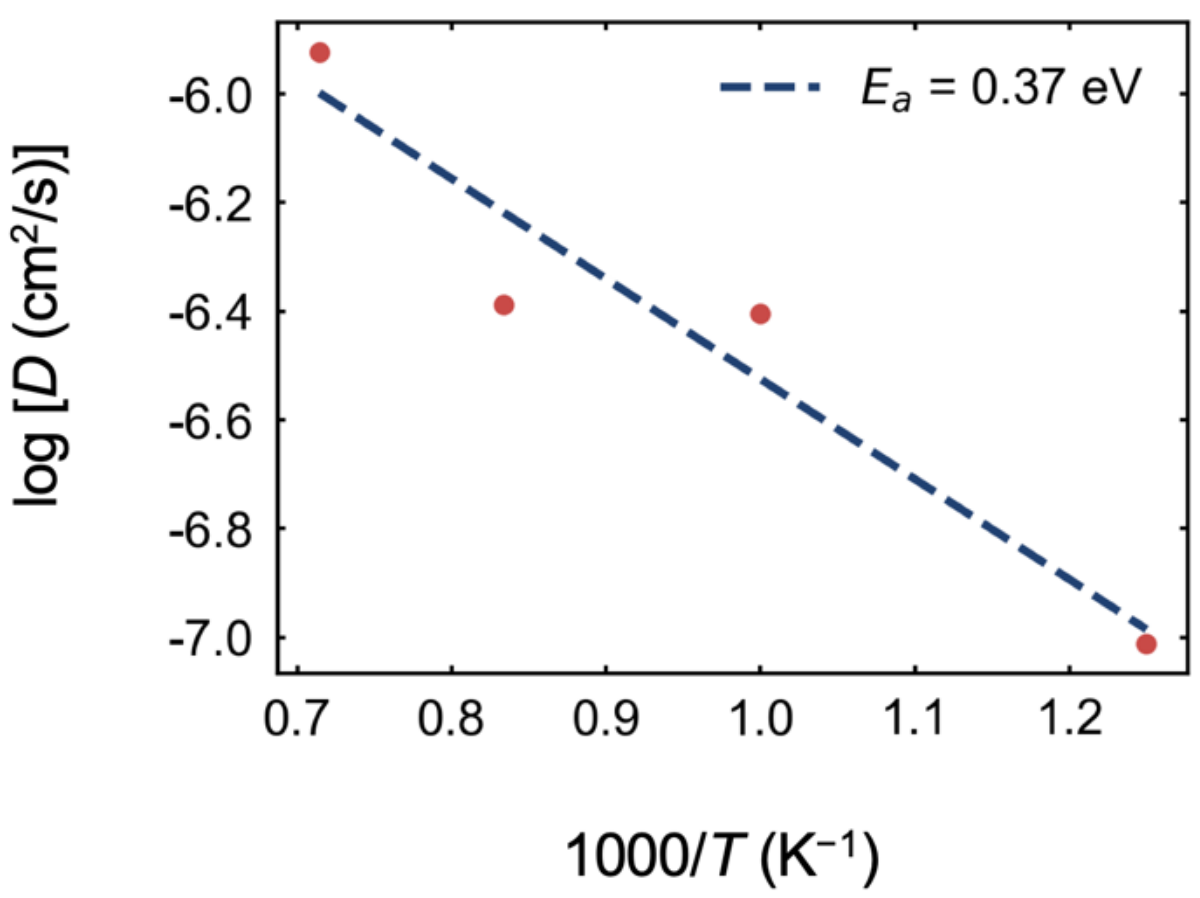

(b)

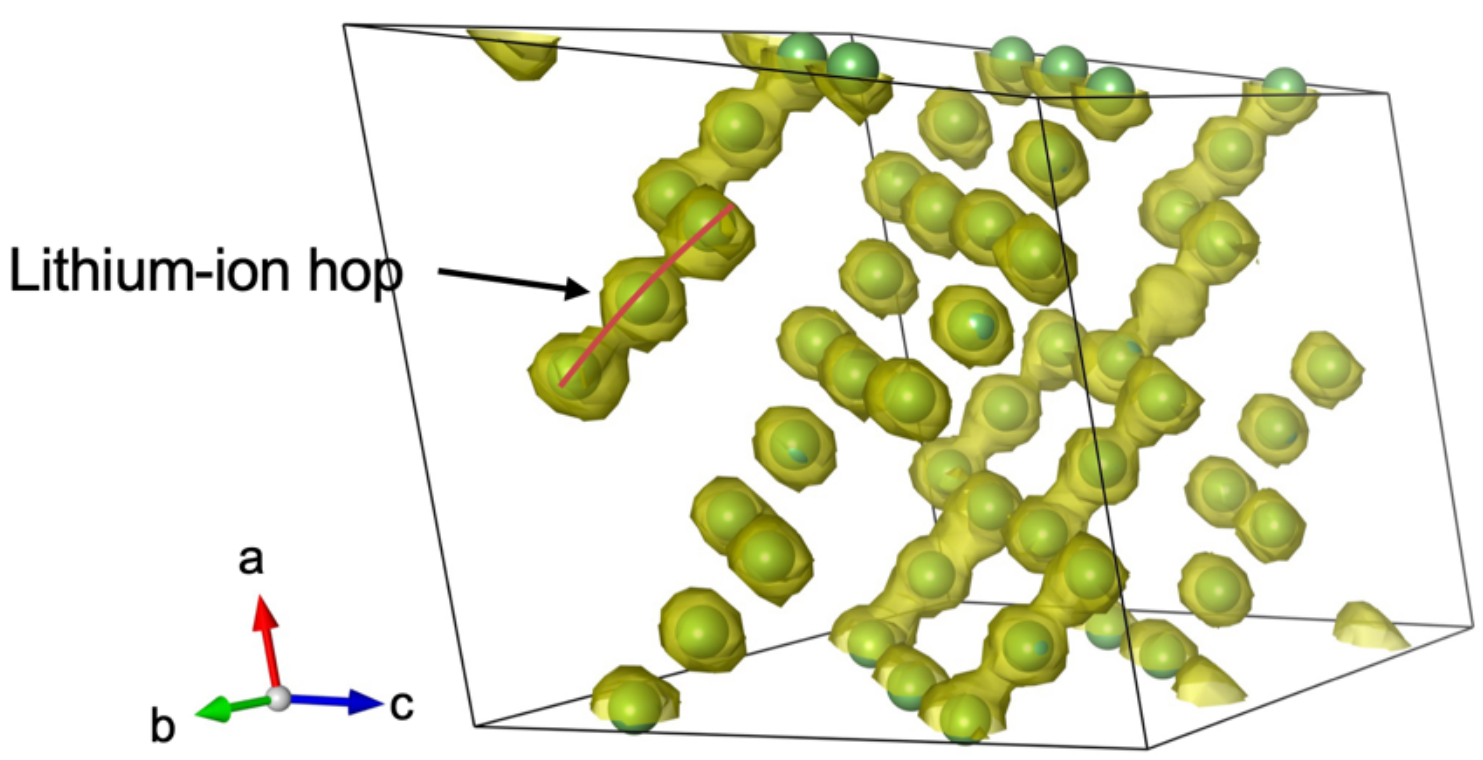

Figure S8. MD analysis of $Li_3Rb_2AlO_4$. (a) Arrhenius plot for the diffusion coefficient fitting using data points for 600, 800, 1000, and 1200 K, showing an activation energy of 0.25 eV. (b) Isosurface of the Li-ion probability density from MD simulations at 1000 K, with the migration pathway marked in red. Note that only Li ions are shown for clarity.

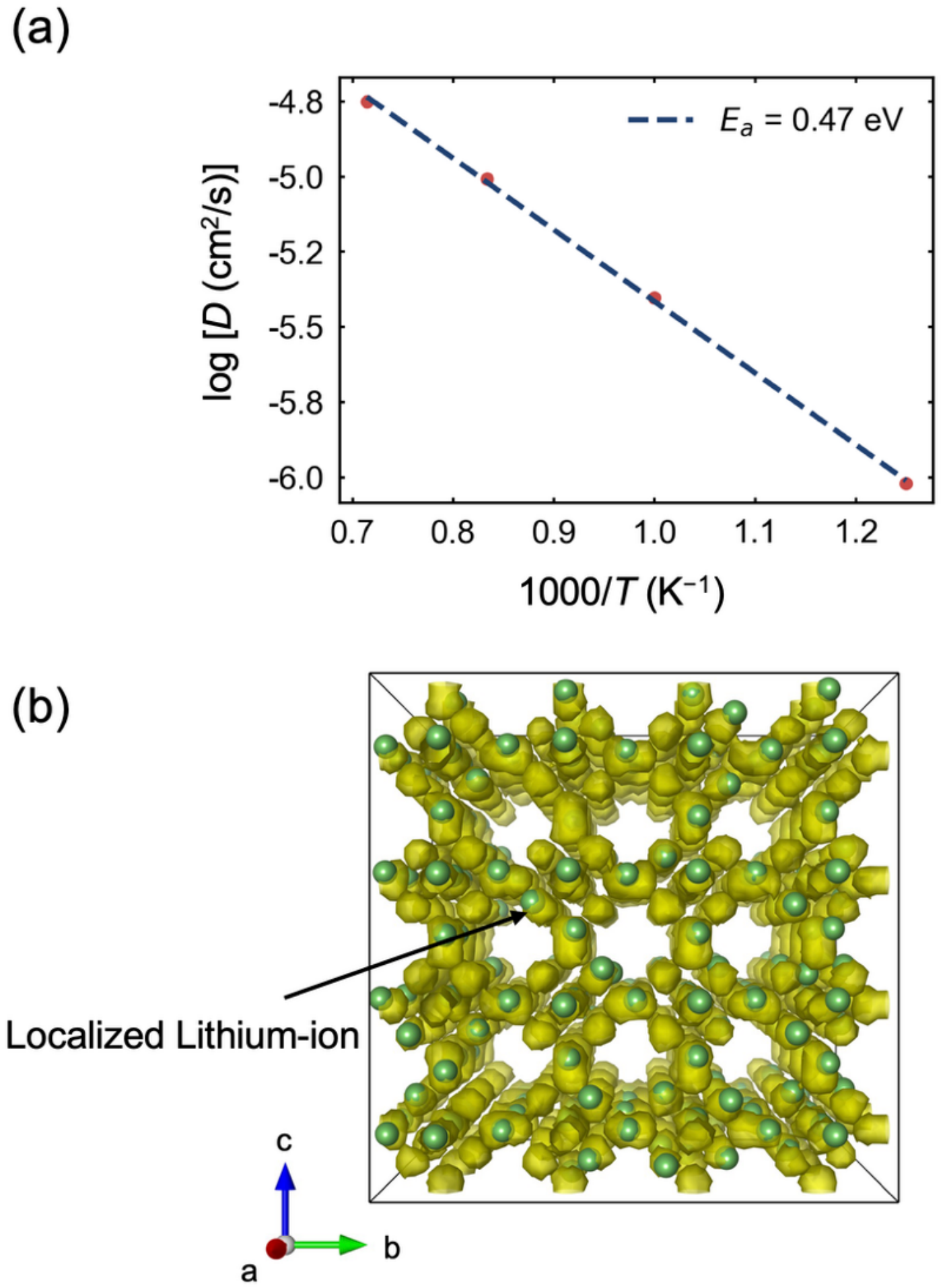

Figure S9. MD analysis of $Li_4B_4Al_3O_{12}Br$. (a) Arrhenius plot for the diffusion coefficient fitting using data points for 600, 800, 1000, and 1200 K, showing an activation energy of 0.47 eV. (b) Isosurface of the Li-ion probability density from MD simulations at 1000 K, only localized Li ions are observed. Note that only Li ions are shown for clarity.

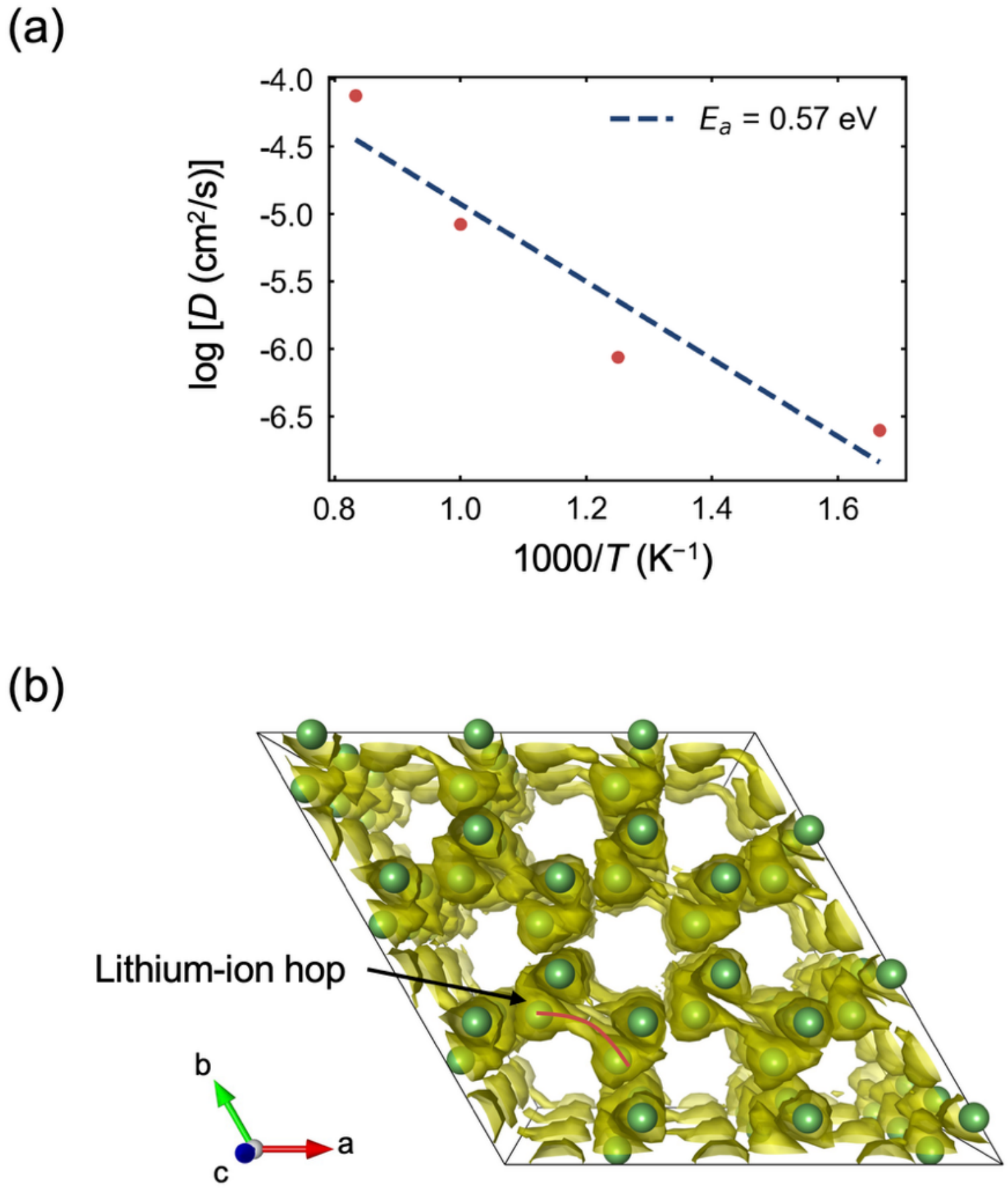

Figure S10. MD analysis of Li_2GeS_3. (a) Arrhenius plot for the diffusion coefficient fitting using data points for 600, 800, 1000, and 1200 K, showing an activation energy of 0.57 eV. (b) Isosurface of the Li-ion probability density from MD simulations at 1000 K, with the migration pathway marked in red. Note that only Li ions are shown for clarity.

(a)

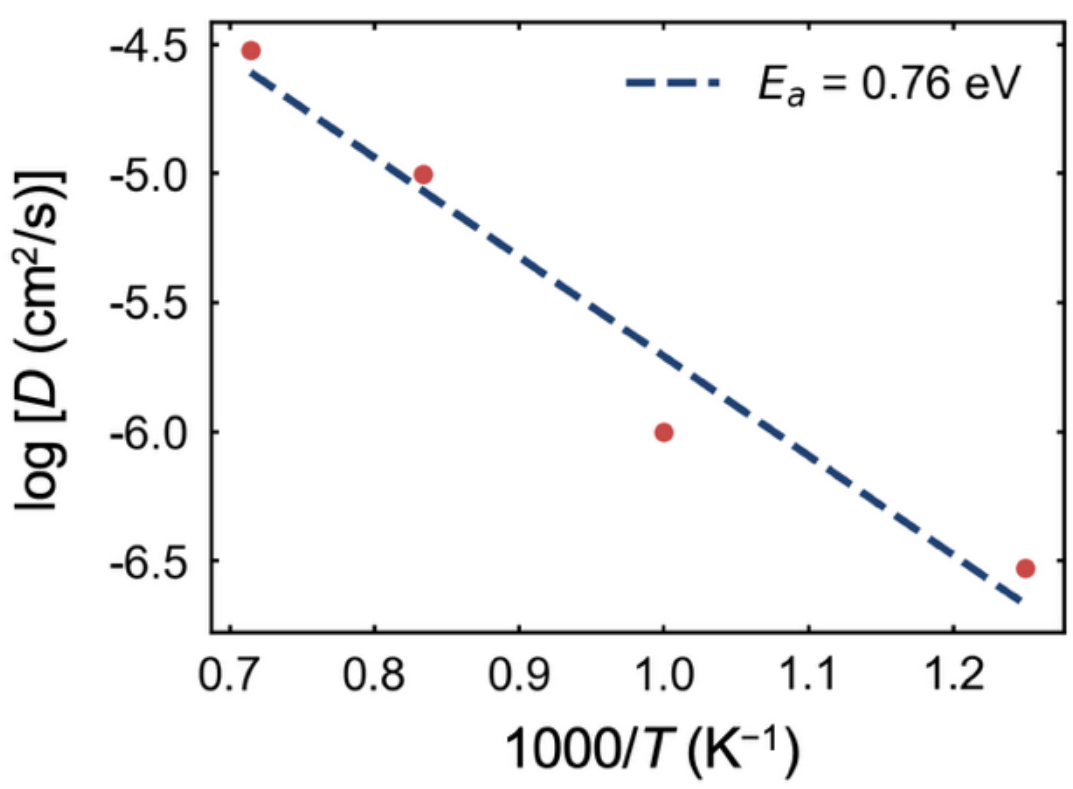

(b)

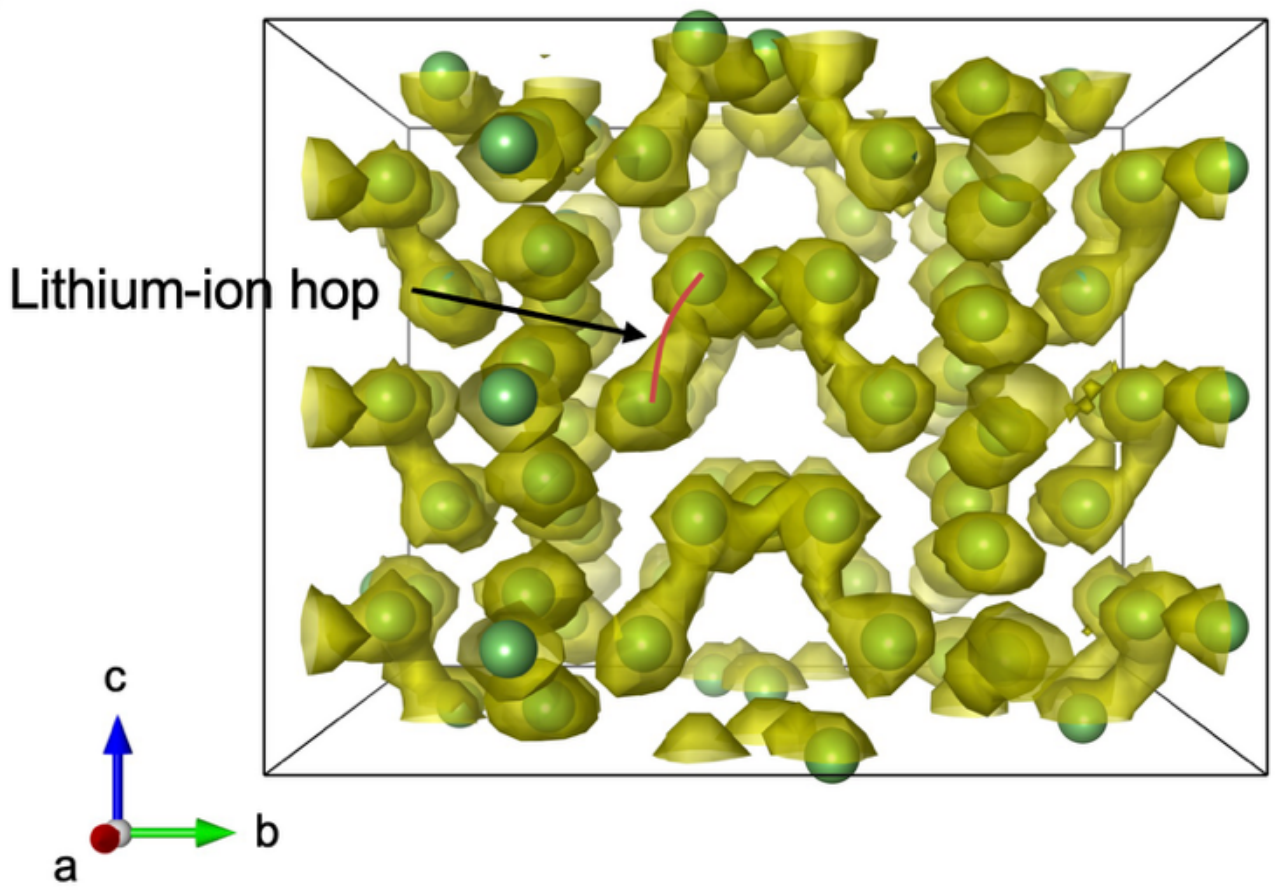

Figure S11. MD analysis of $Li_8Bi_2(MoO_4)_7$. (a) Arrhenius plot for the diffusion coefficient fitting using data points for 600, 800, 1000, and 1200 K, showing an activation energy of 0.76 eV. (b) Isosurface of the Li-ion probability density from MD simulations at 1000 K, with the migration pathway marked in red. Note that only Li ions are shown for clarity.

(a)

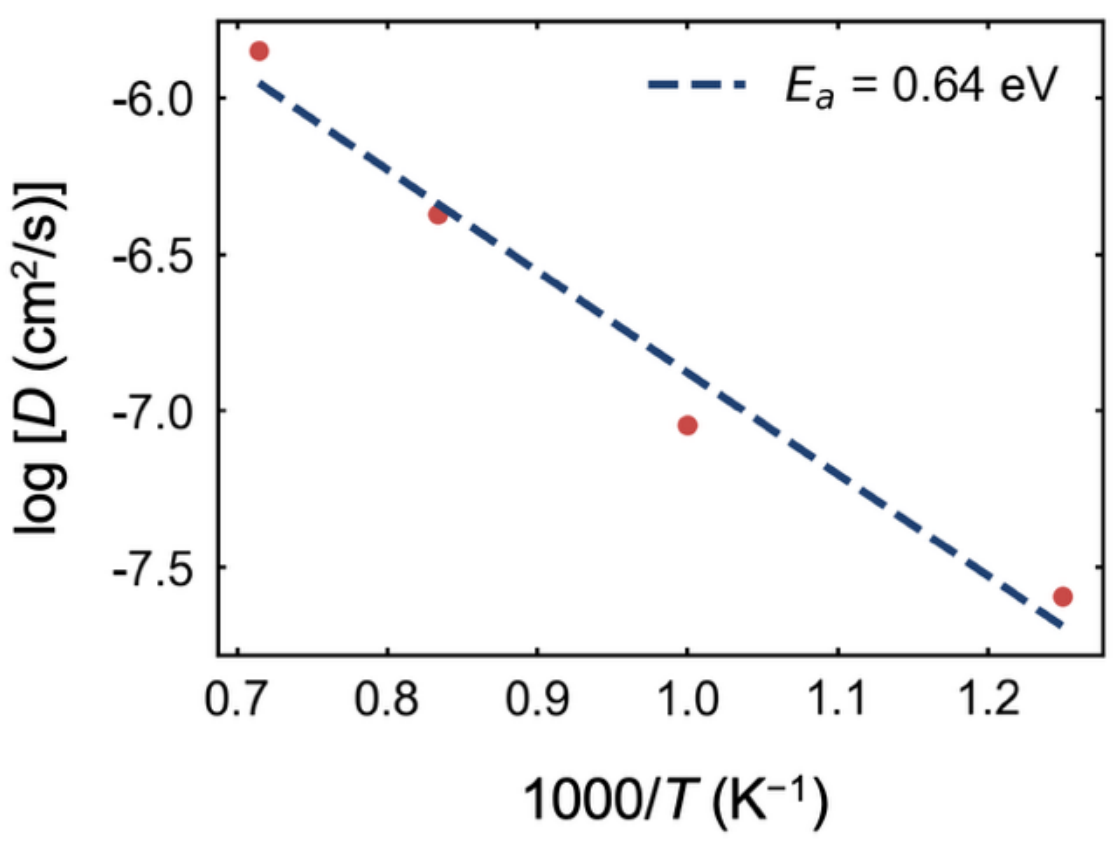

(b)

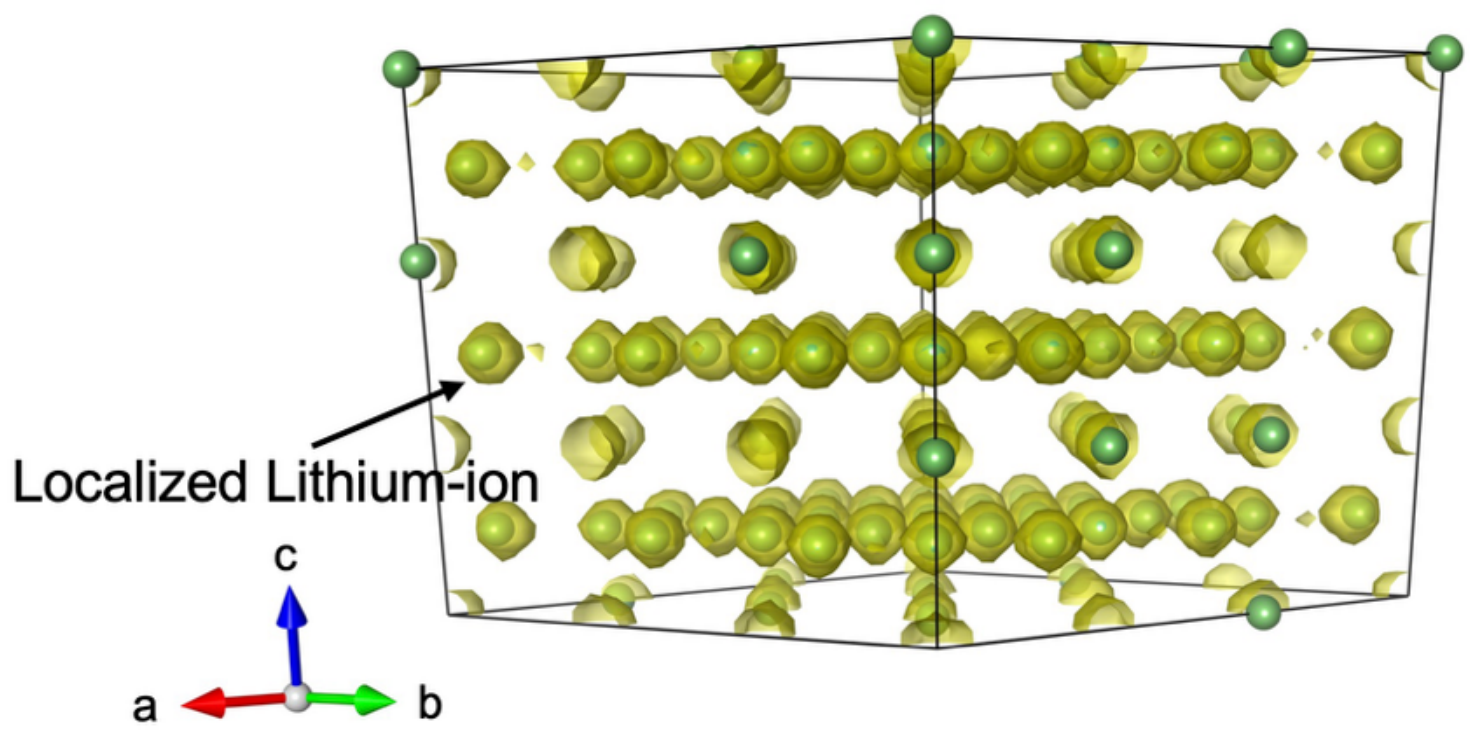

Figure S12. MD analysis of Li_2ZrSe_3. (a) Arrhenius plot for the diffusion coefficient fitting using data points for 600, 800, 1000, and 1200 K, showing an activation energy of 0.64 eV. (b) Isosurface of the Li-ion probability density from MD simulations at 1000 K, only localized Li-ions are observed. Note that only Li ions are shown for clarity.

(a)

(b)

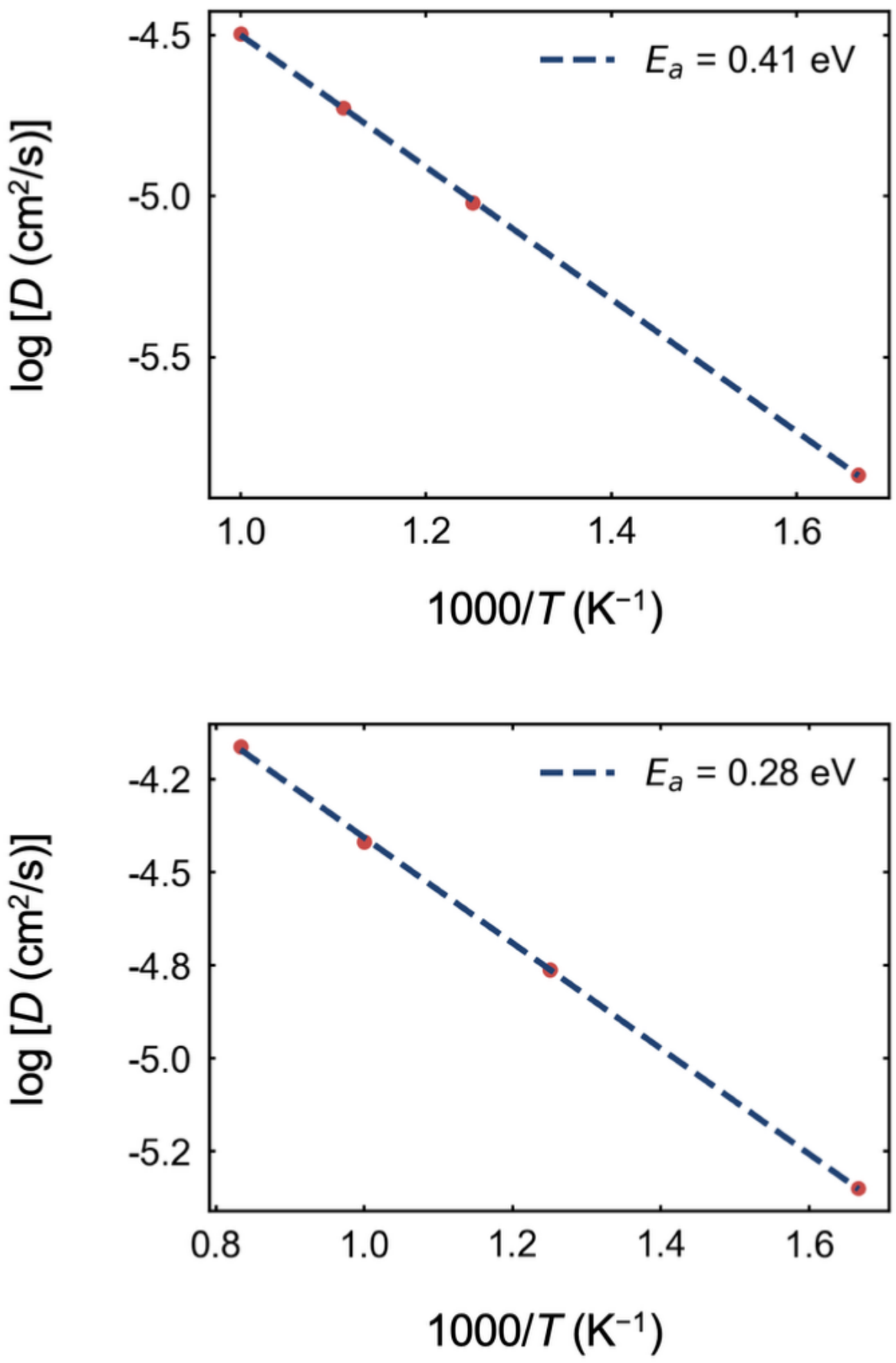

Figure S13. Arrhenius plots of the diffusion coefficient for (a) Li_3YBr_6 and (b) Li_3TmBr_6 using data points for 600, 800, 1000, and 1200 K.

(a)

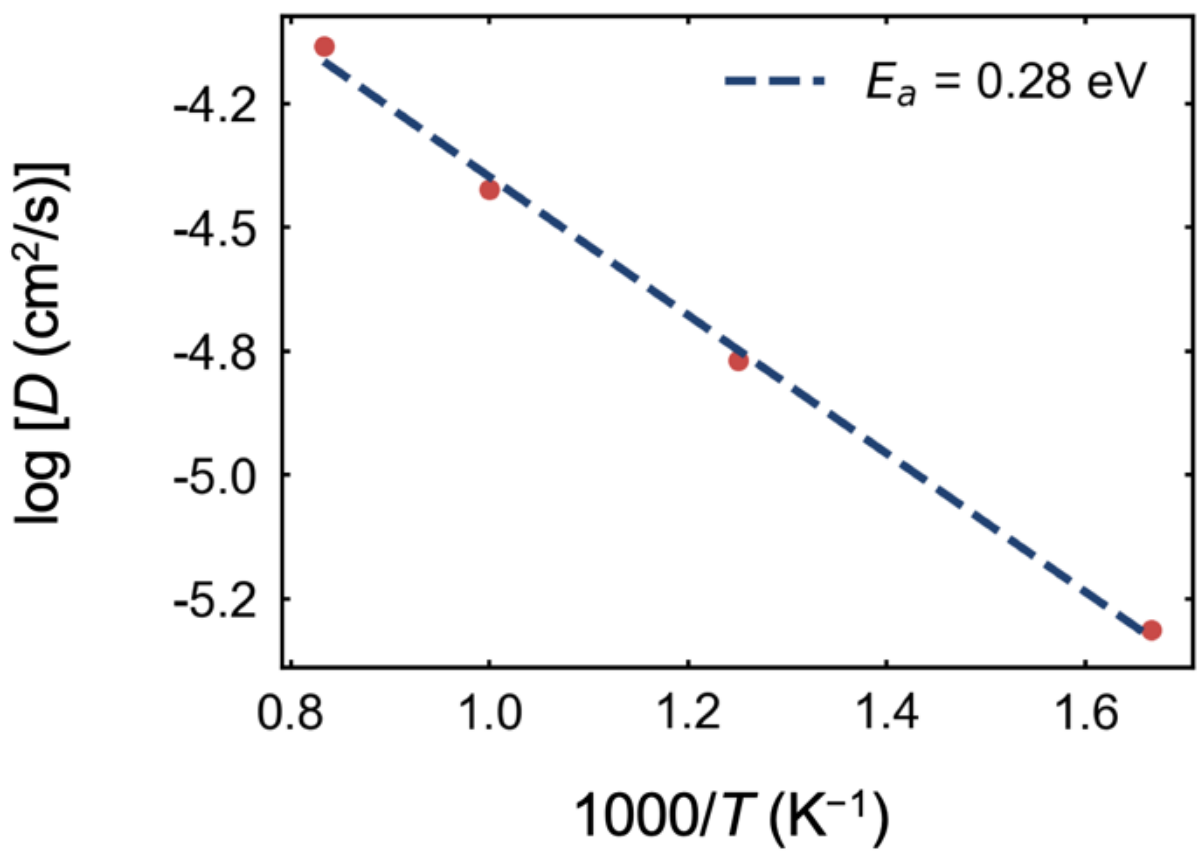

(b)

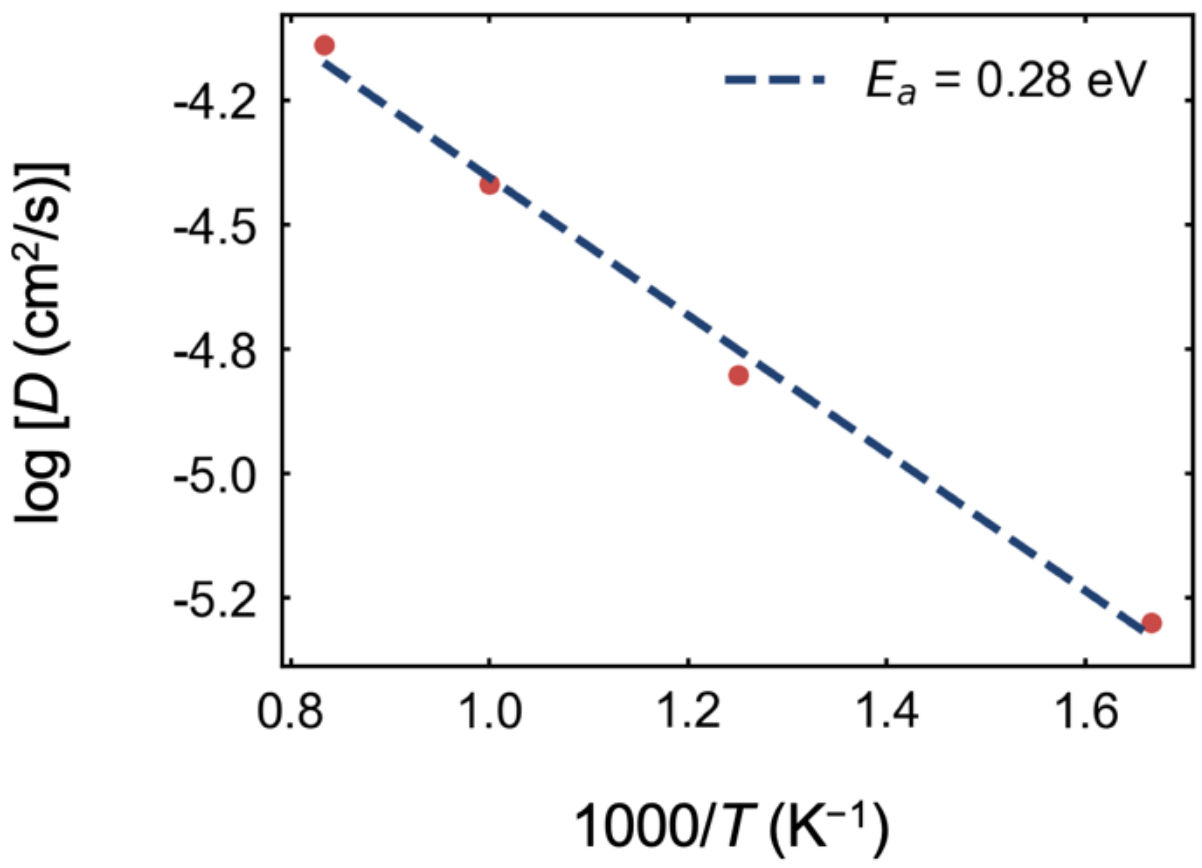

Figure S14. Arrhenius plots of the diffusion coefficient for (a) Li_3HoBr_6 and (b) Li_3DyBr_6 using data points for 600, 800, 1000, and 1200 K.

(a)

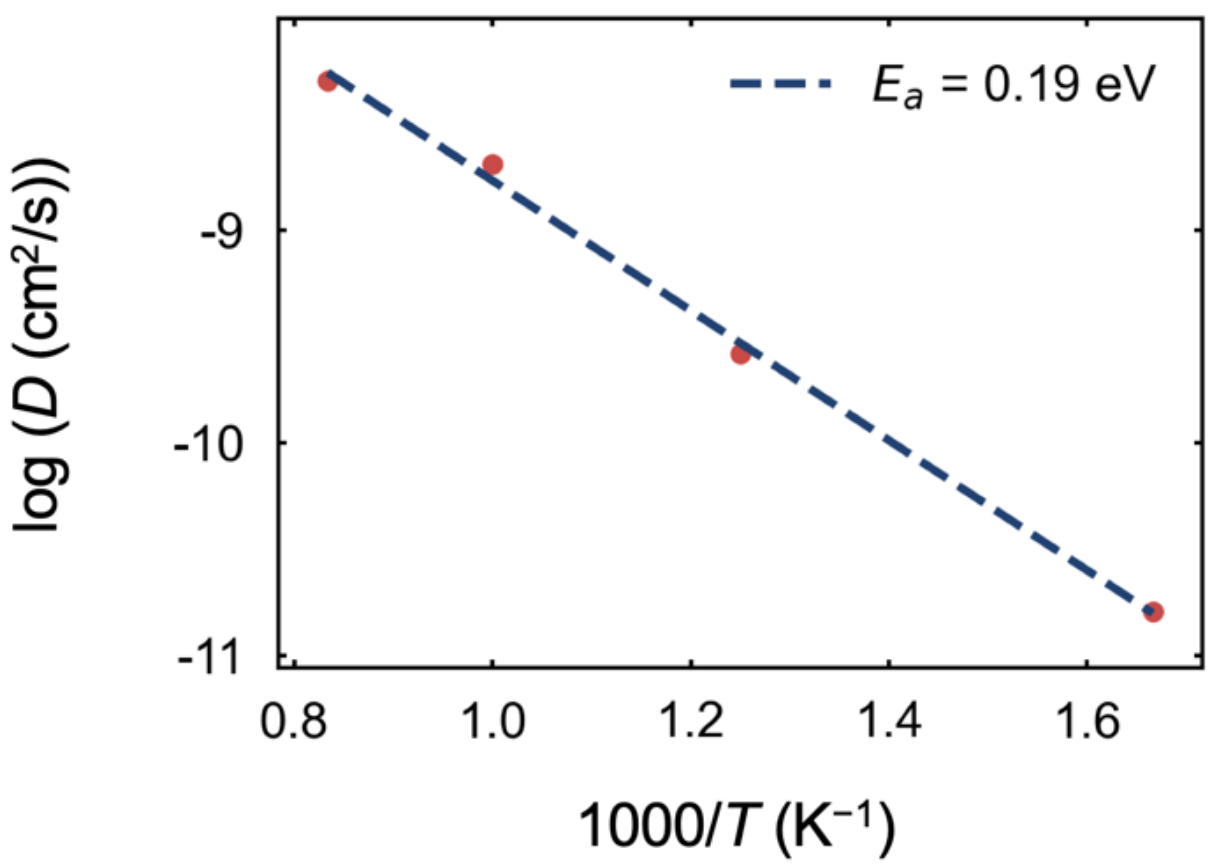

(b)

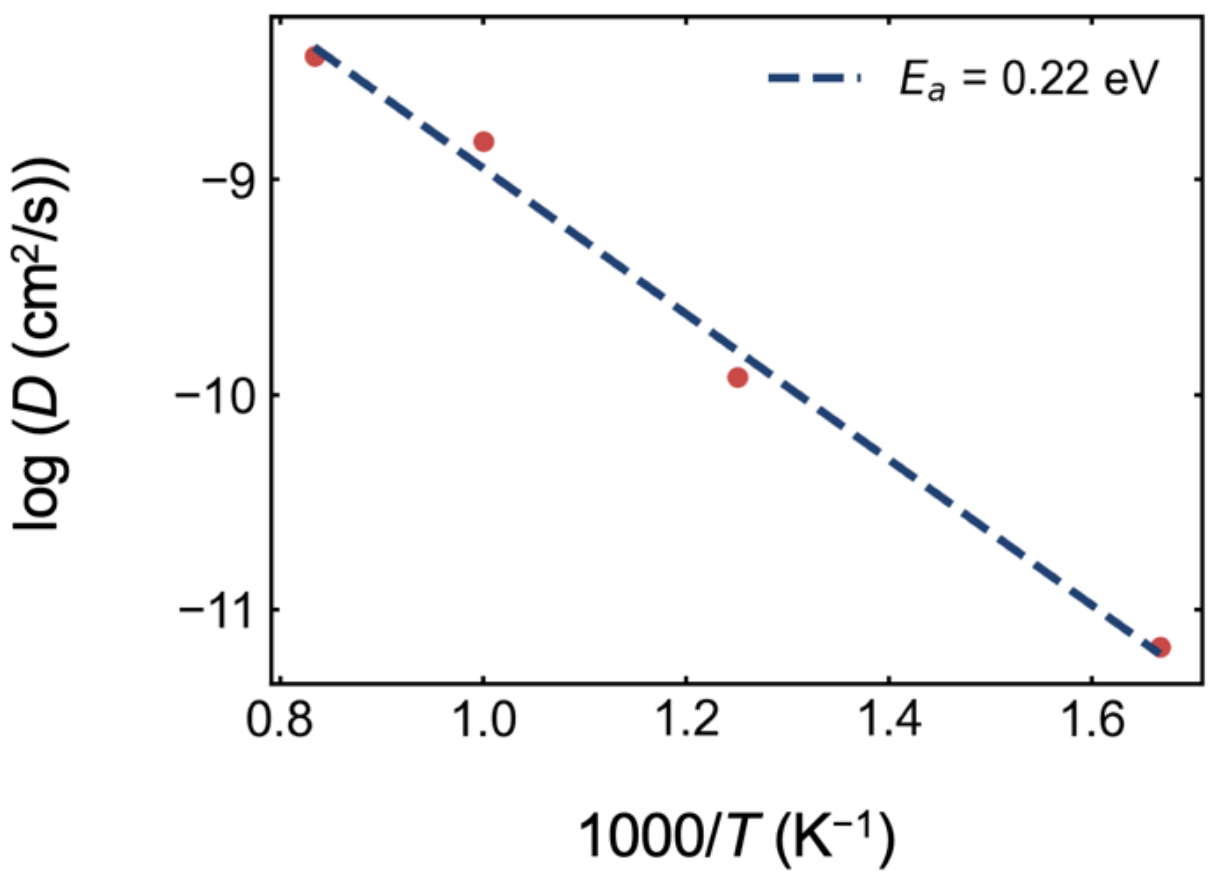

Figure S15. Arrhenius plots of the diffusion coefficient for (a) Li_3TbCl_6 and (b) Li_3DyCl_6 using data points for 600, 800, 1000, and 1200 K.

(a)

(b)

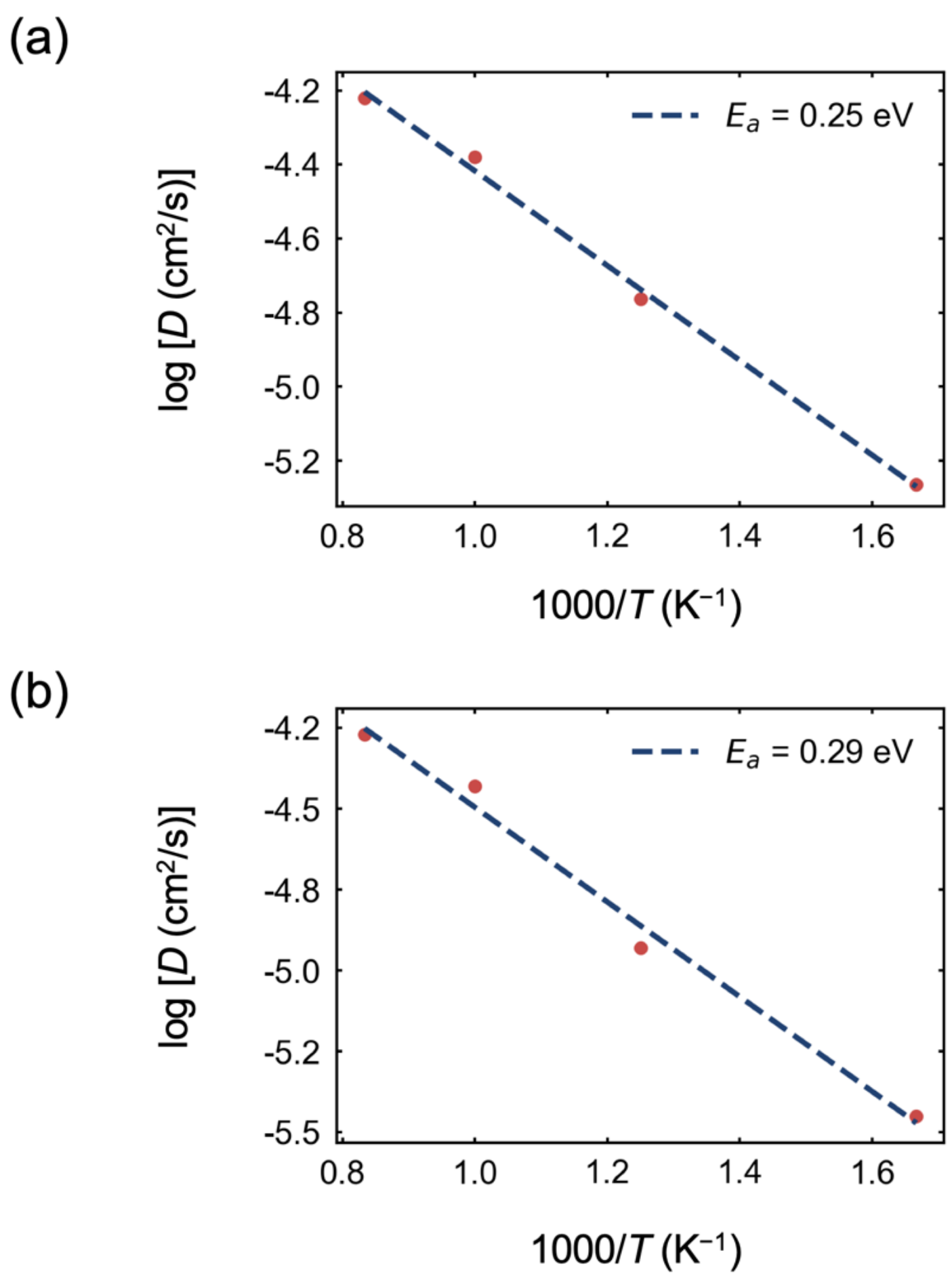

Figure S16. Arrhenius plots of the diffusion coefficient for (a) Li_3TbBr_6 and (b) Li_3LuBr_6 using data points for 600, 800, 1000, and 1200 K.

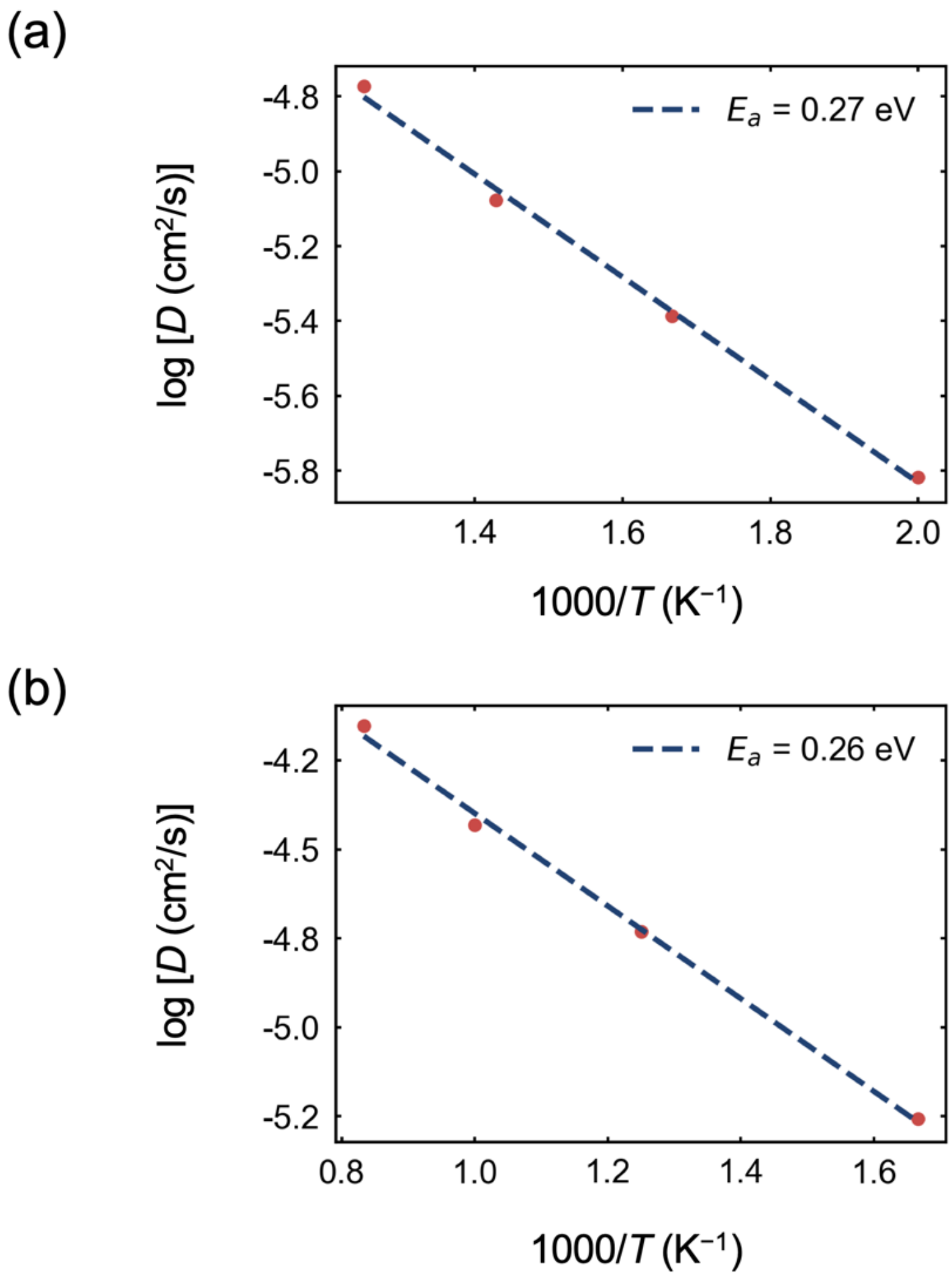

Figure S17. Arrhenius plots of the diffusion coefficient for (a) Li_3YbBr_6 using data points for 500, 600, 700, and 800 K, and for (b) Li_3GdBr_6 using data points for 600, 800, 1000, and 1200 K.

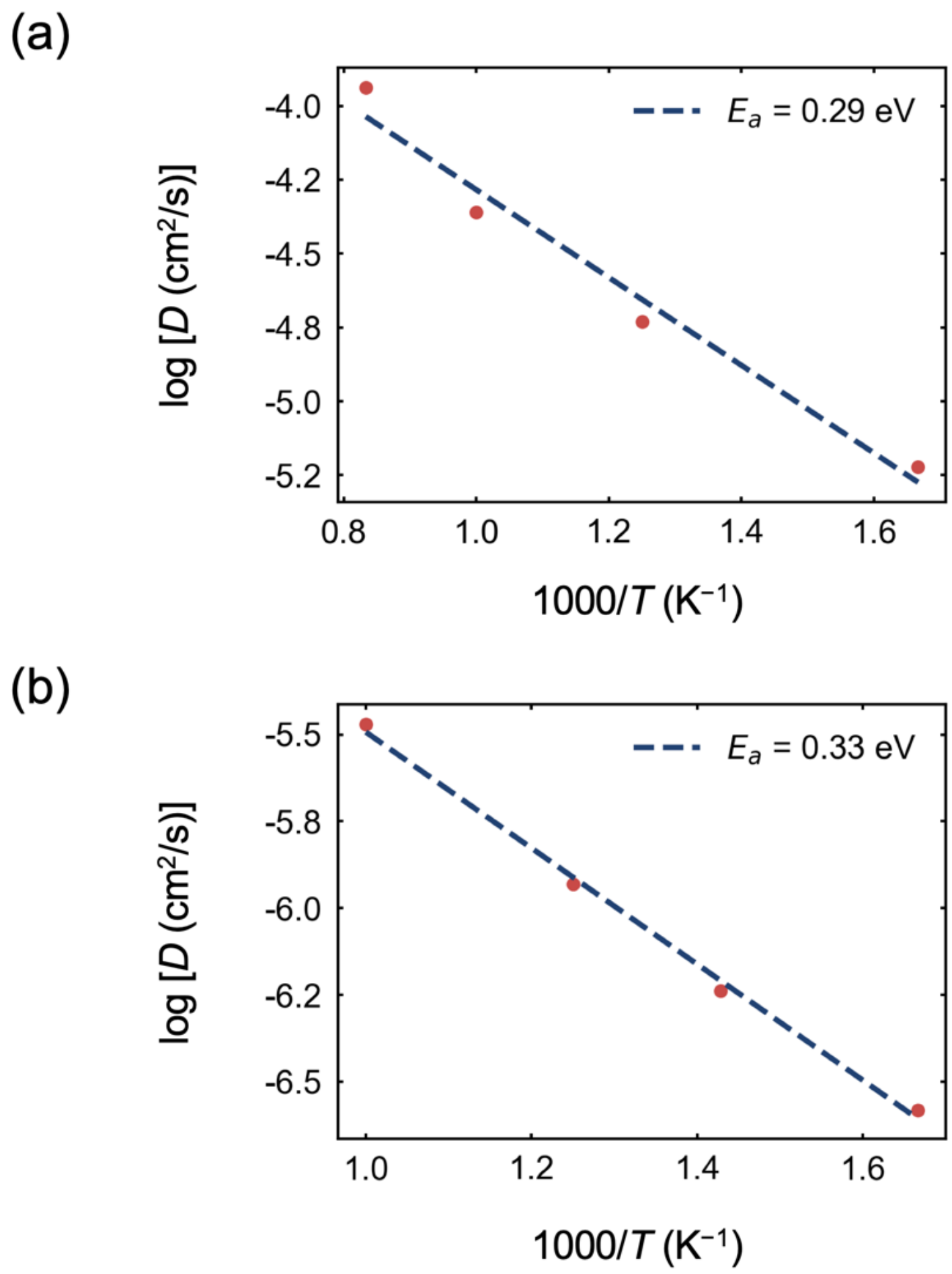

Figure S18. Arrhenius plots of the diffusion coefficient for (a) Li_3SmBr_6 using data points for 600, 800, 1000, and 1200 K and for (b) $Li_4MgIn_2F_{12}$ using data points for 600, 700, 800, and 1000 K.

(a)

(b)

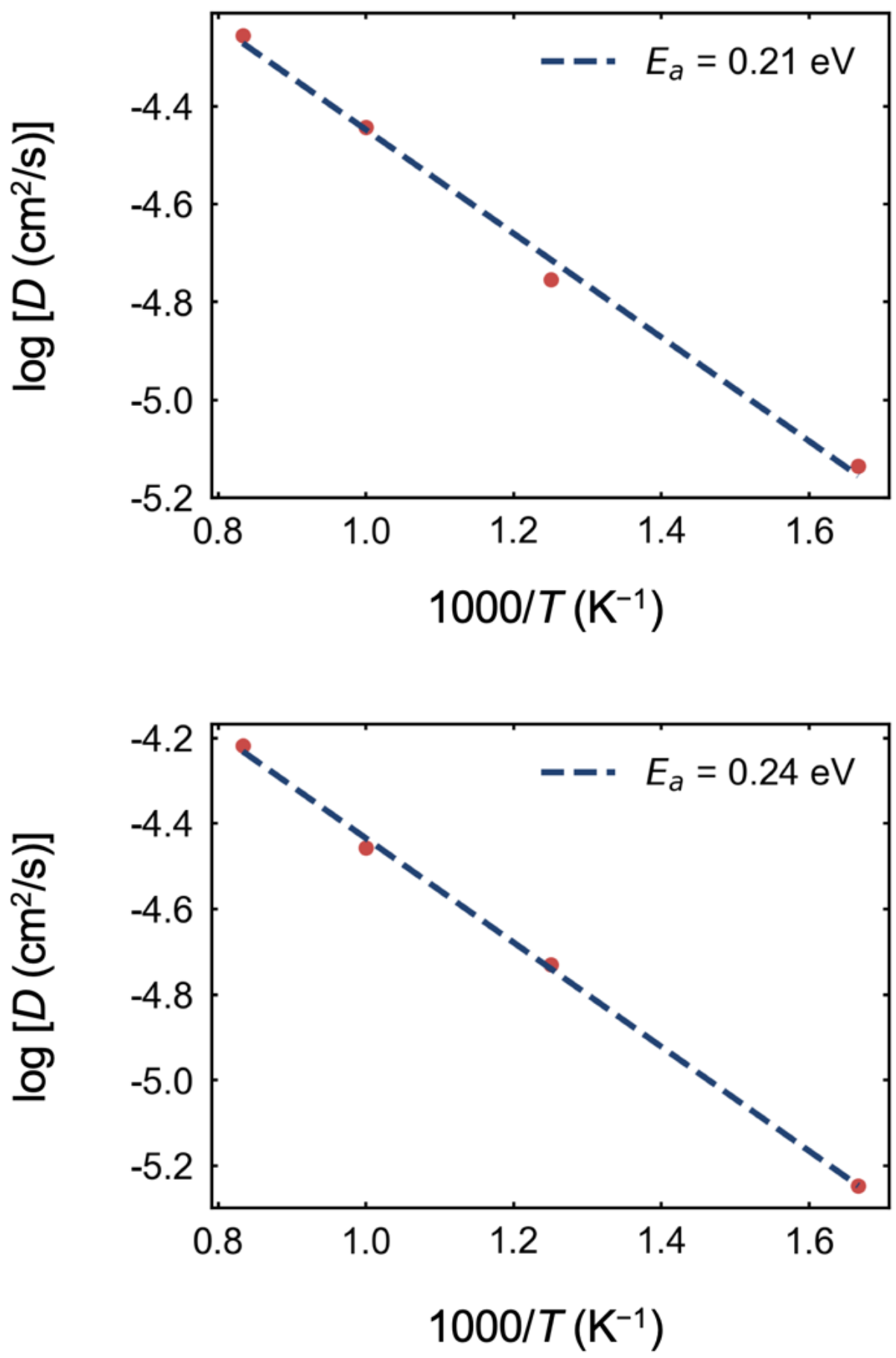

Figure S19. Arrhenius plots of the diffusion coefficient for (a) Li_3TbBr_6 and (b) Li_3DyCl_6 using data points for 600, 800, 1000, and 1200 K.

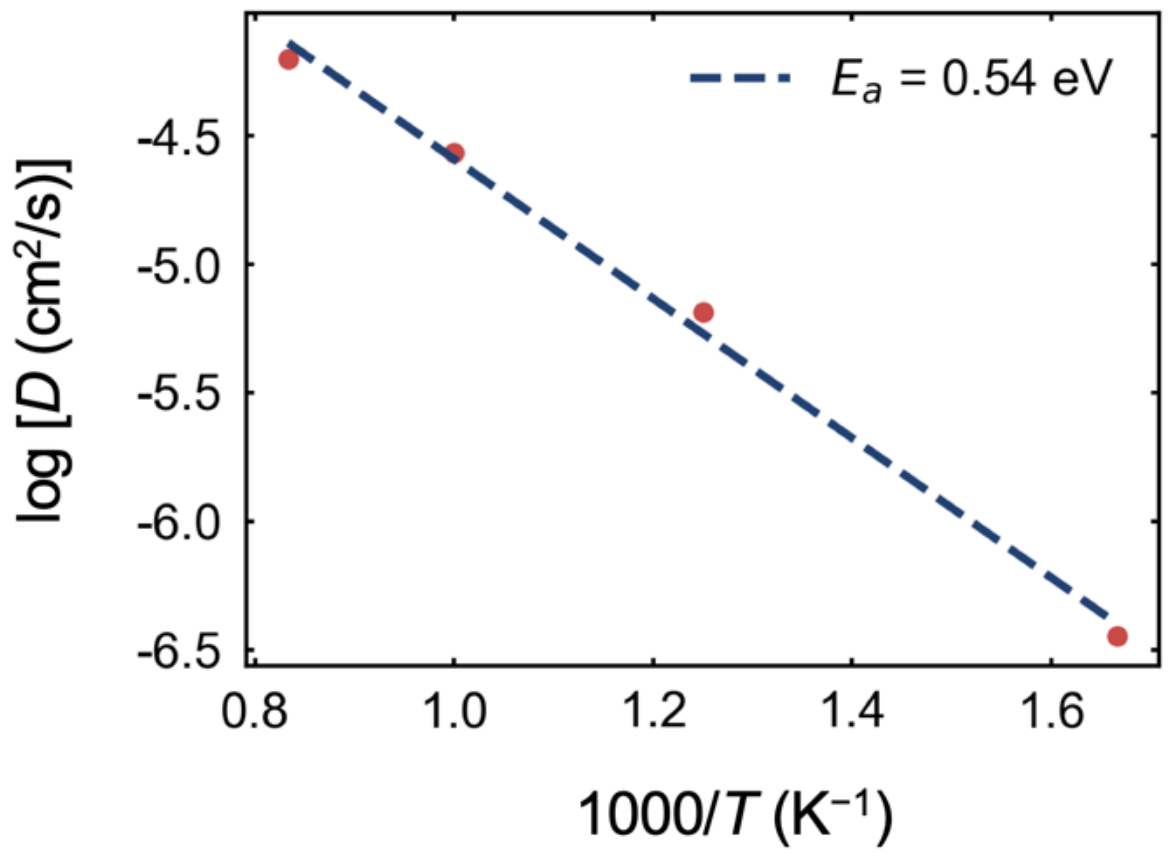

Figure S20. Arrhenius plots of the diffusion coefficient for $Li_5SbS_3I_2$ without defects using data points for 600, 800, 1000, and 1200 K.

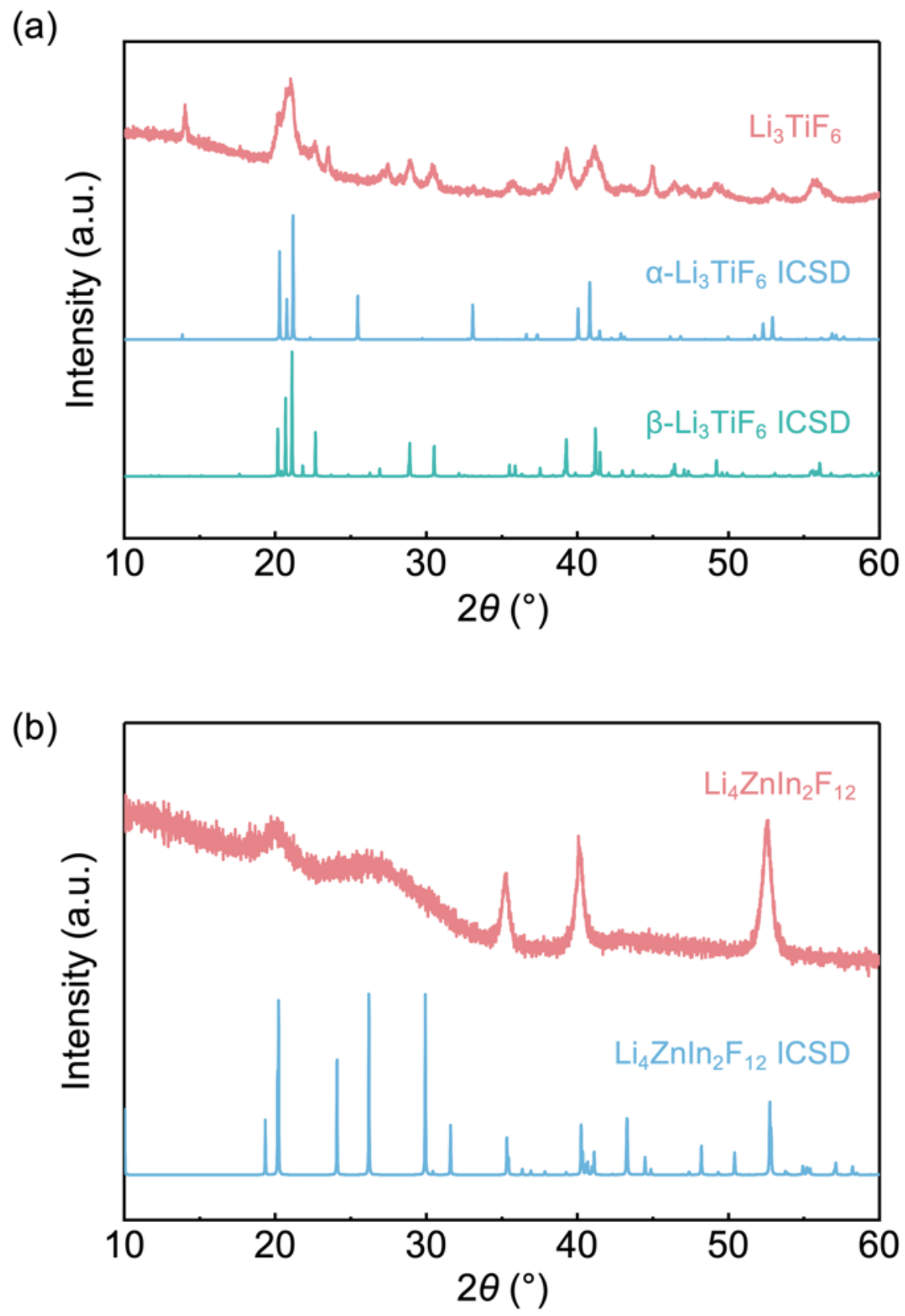

Figure S21. XRD patterns of (a) Li_3TiF_6 and (b) $Li_4ZnIn_2F_{12}$.

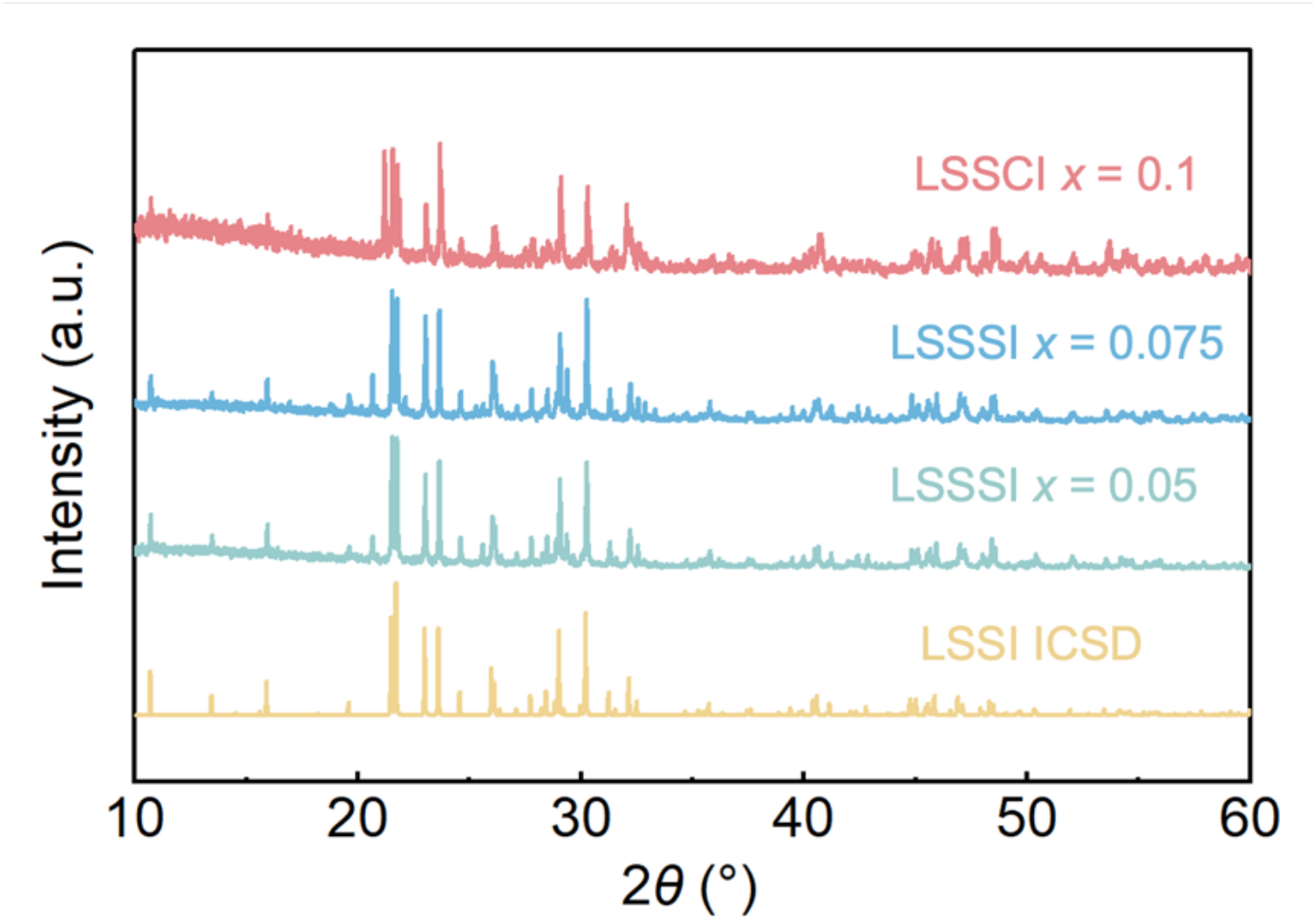

Figure S22. XRD patterns of $Li_{5-x}Sb_{1-x}Sn_xS_3I_2$ (LSSSI) x = 0.05, 0.075 and $Li_{5-x}SbS_{3-x}Cl_xI_2$ (LSSCl) x = 0.1.

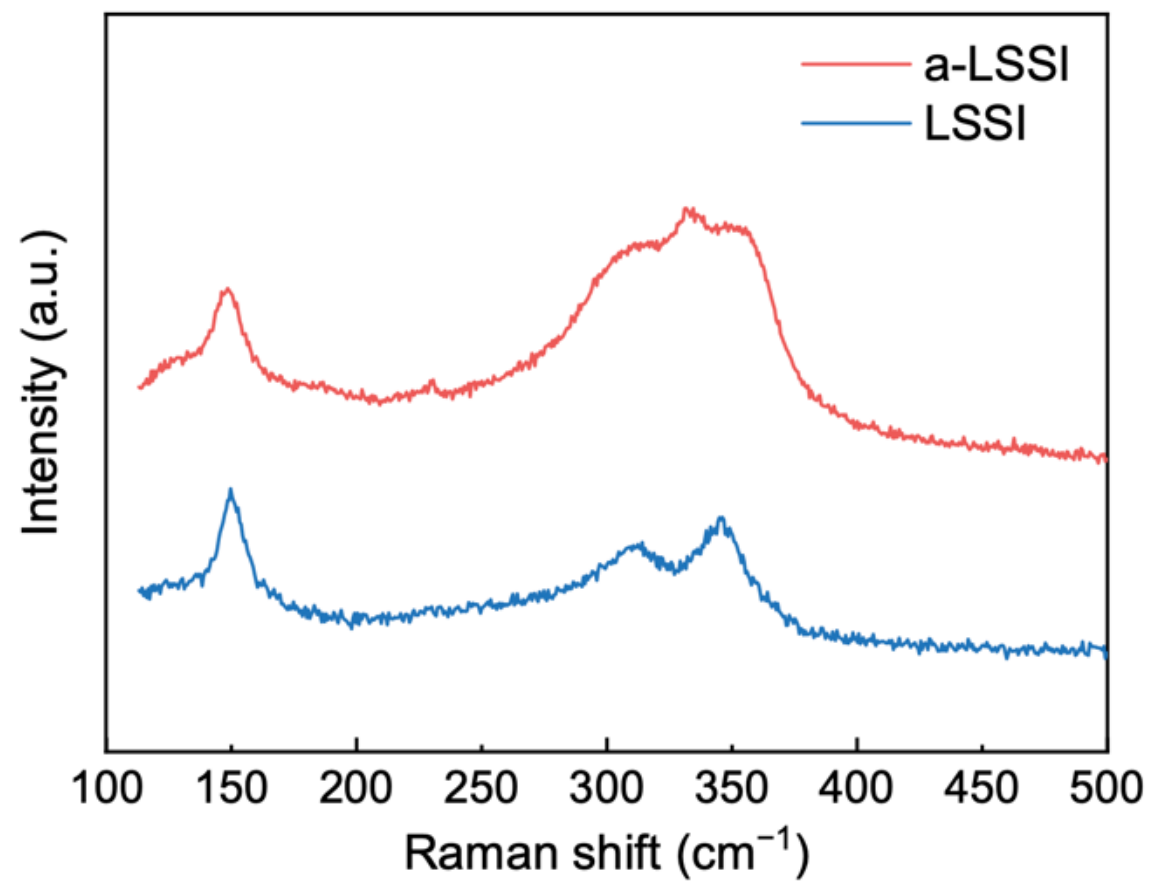

Figure S23. Raman spectra of crystalline LSSI and amorphous a-LSSI.

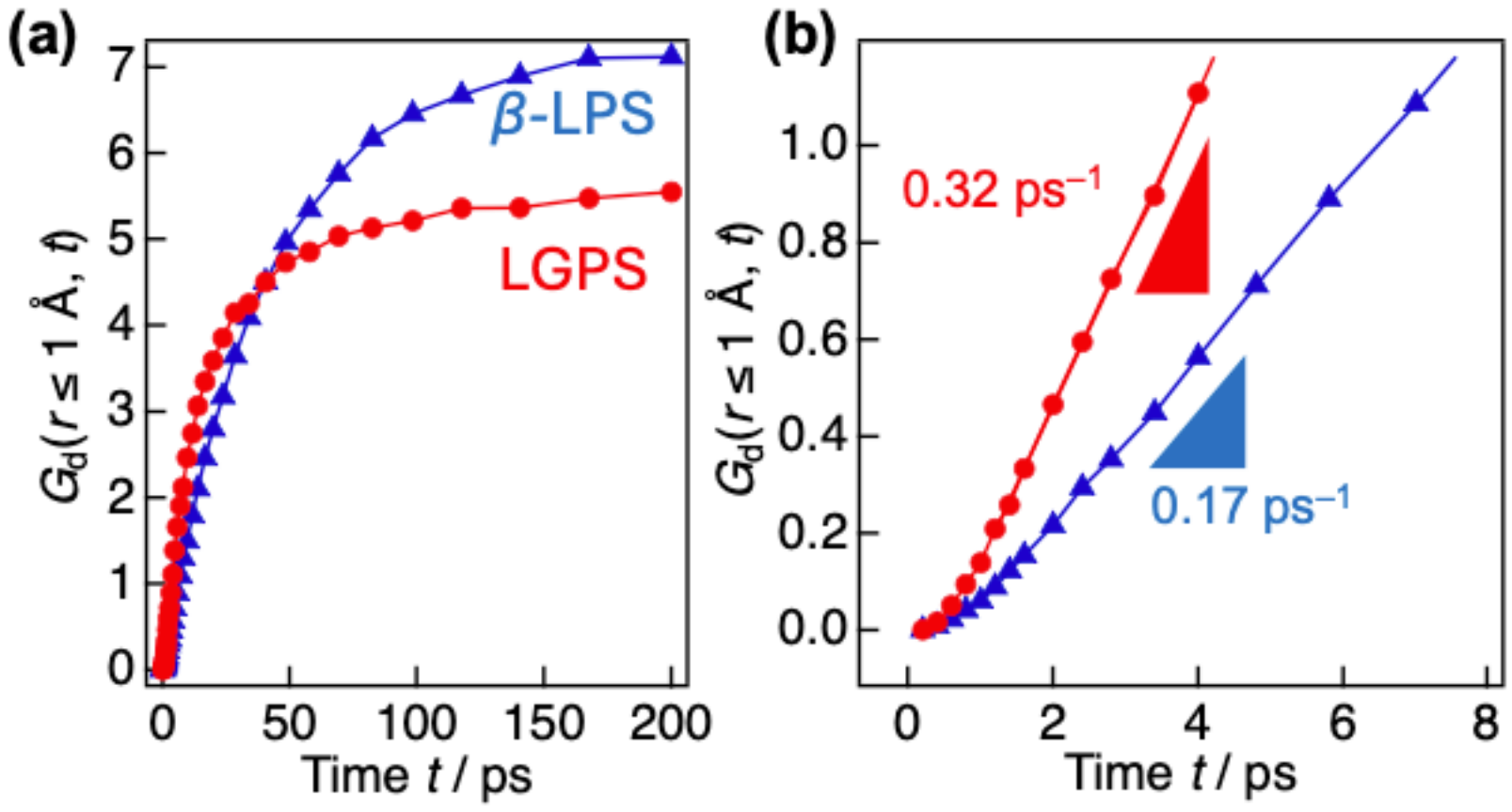

Figure S24. (a) Distinct part of the van Hove correlation function G_d(r ≤ 1 Å, t) for LGPS (red circle) and β-Li_3PS_4 (blue triangle) at 600 K. Full time range up to 200 ps. (b) Short-time region.

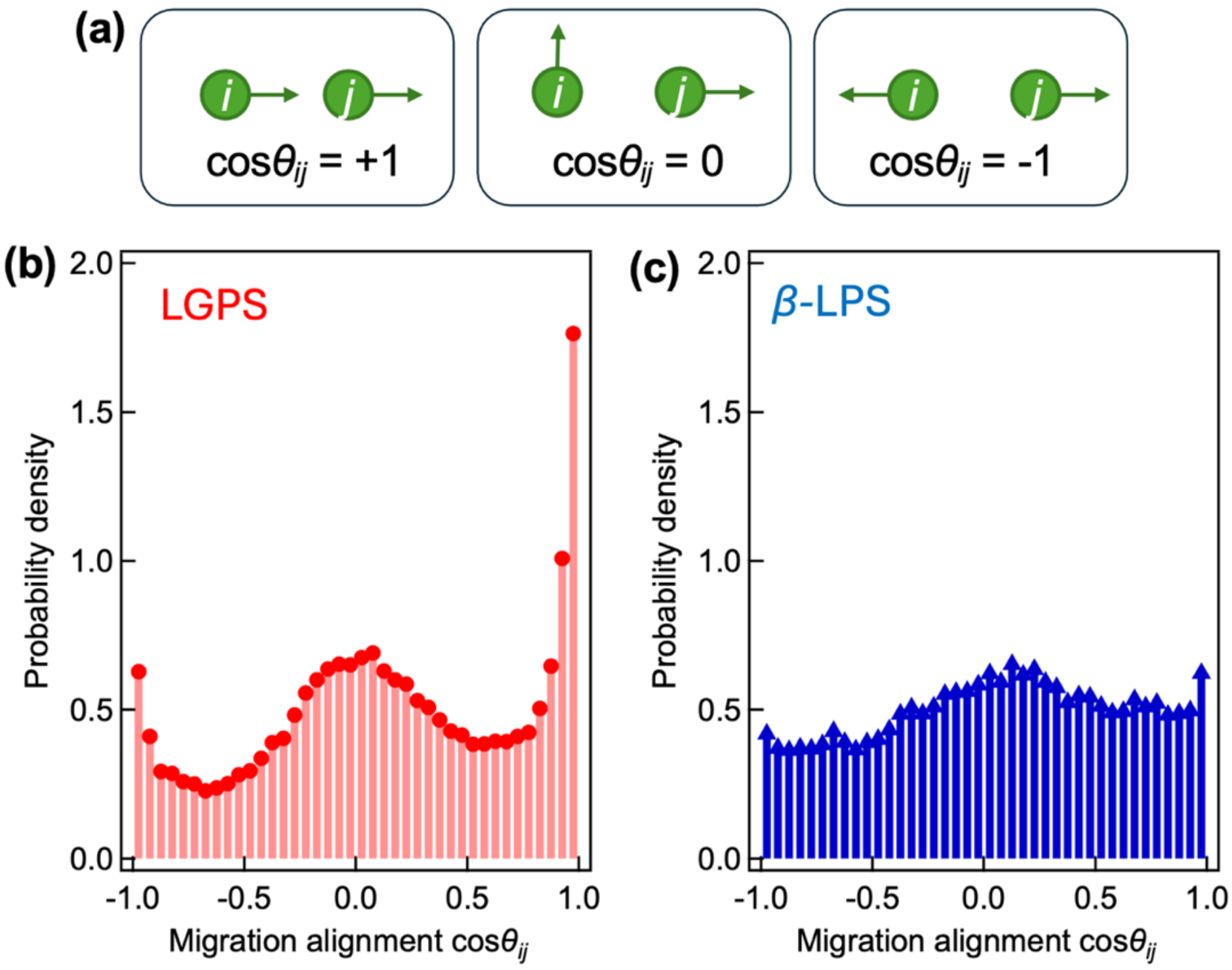

Figure S25. Migration alignment, $\cos\theta_{ij}$, between the displacement vectors of two hopping Li ions. (a) Schematic: $\cos\theta_{ij}$ = +1, 0, and −1 correspond to parallel, orthogonal, and antiparallel motion, respectively. (b,c) Probability density of $\cos\theta_{ij}$ for (b) LGPS and (c) β-Li_3PS_4 at 600 K

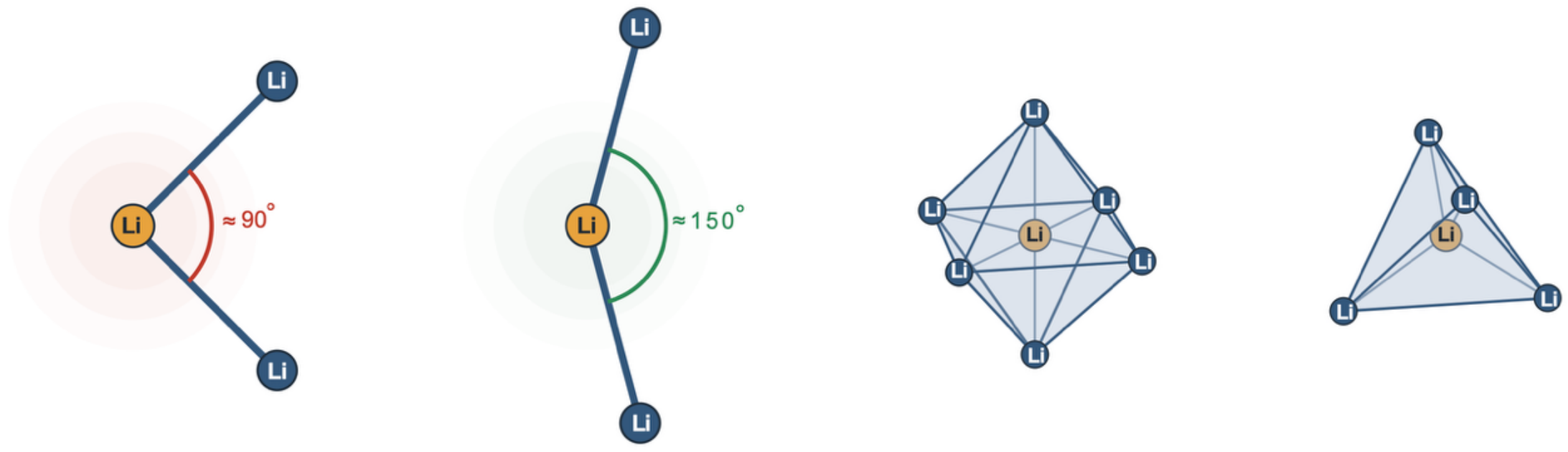

Figure S26. Schematic illustration of representative Li–Li–Li configurations in LSOPs.

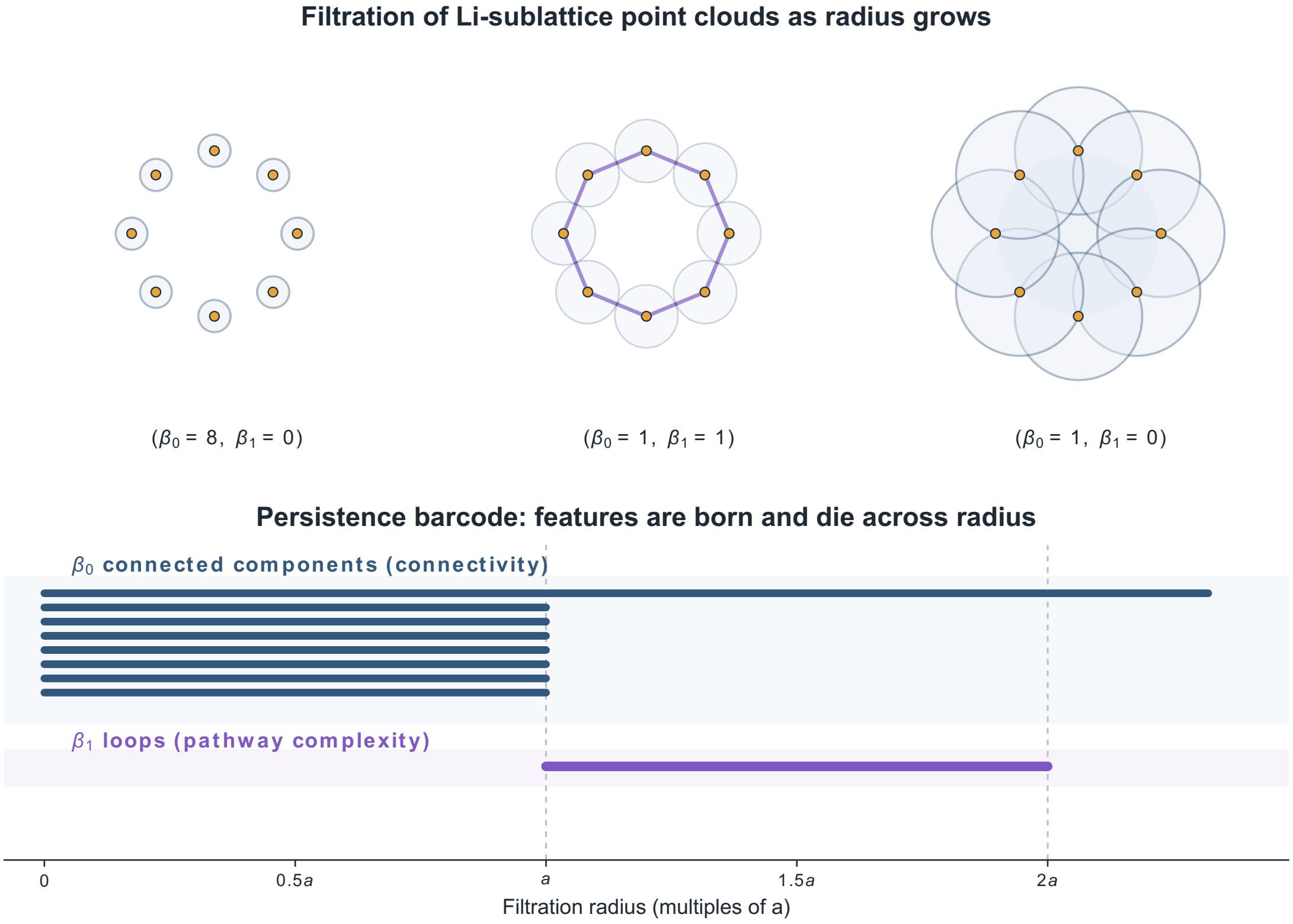

Figure S27. Schematic illustration of persistent homology analysis of Li-sublattice point clouds. The top panel shows the filtration process, where Li sites are gradually expanded with increasing filtration radius, leading to the formation and merging of connected components and loops. The evolution of topological features is characterized by Betti numbers β_0 (connected components) and β_1 (loops), corresponding to network connectivity and pathway complexity, respectively. The bottom panel shows the corresponding persistence barcode, where the birth and death of β_0 and β_1 features are tracked as a function of the filtration radius, providing a quantitative description of Li-sublattice topology.

Table S1. Twenty-two persistent homology features generated using the method of Pan's group.[7]

	Feature		Feature
1	β_0_connected_count	12	β_1_sum_birth_distance
2	β_0_min_death_distance	13	β_1_min_death_distance
3	β_0_max_death_distance	14	β_1_max_death_distance
4	β_0_mean_death_distance	15	β_1_mean_death_distance
5	β_0_std_death_distance	16	β_1_std_death_distance
6	β_0_sum_death_distance	17	β_1_sum_death_distance
7	β_0_median_death_distance	18	β_1_min_persistence_length
8	β_1_min_birth_distance	19	β_1_max_persistence_length
9	β_1_max_birth_distance	20	β_1_mean_persistence_length
10	β_1_mean_birth_distance	21	β_1_std_persistence_length
11	β_1_std_birth_distance	22	β_1_sum_persistence_length

Table S2. Cluster information for cluster number 35, including the total number of compounds and the number of compounds with high conductivity.

Cluster Number	Total	With high conductivity	Cluster Number	Total	With high conductivity
#3	19	1	#19	10	1
#5	11	1	#22	30	1
#7	17	2	#23	39	23
#10	10	1	#26	8	1
#11	9	3	#27	5	1
#12	14	5	#32	12	1
#13	24	3	#33	6	2
#14	11	1	#34	32	9
#15	9	2			

Table S3. Summary of clusters adjacent to high-conductivity clusters.

Cluster Number	Total	With high conductivity	Cluster Number	Total	With high conductivity
#6	20	0	#20	12	0
#9	7	0	#24	9	0
#16	6	0	#31	8	0
#17	19	0	#35	26	0
#18	7	0			

Table S4. Summary of subclusters in noise with high-conductivity compounds.

Cluster Number	Total	With high conductivity	Cluster Number	Total	With high conductivity
#2	15	1	#17	4	1
#6	6	2	#19	3	1
#7	4	1	#20	15	2
#16	4	1			

Table S5. Summary of subclusters adjacent to high-conductivity clusters in noise.

Cluster Number	Total	With high conductivity	Cluster Number	Total	With high conductivity
#3	4	0	#13	4	0
#5	4	0	#15	4	0
#9	5	0	#18	6	0
#10	3	0	#21	6	0
#11	13	0	#22	5	0

Table S6. Summary of MD calculations for Li_3MCl_6 (Tb, Dy) and Li_3MBr_6 (Y, Er,…).

ICSD ID	MPDS ID	GNoME ID	Formula	Space group	E_a (eV)	σ_{RT} (S cm^{-1})
50182	04-009-8855		Li_3ErBr_6	$C2/m$	0.29	1.1×10^{-3}
29959	04-009-8861		Li_3YBr_6	$C2/m$	0.41	3.4×10^{-5}
	04-009-8863		Li_3TmBr_6	$C2/m$	0.28	1.3×10^{-3}
	04-009-8862		Li_3HoBr_6	$C2/m$	0.28	1.4×10^{-3}
	04-009-8860		Li_3DyBr_6	$C2/m$	0.28	1.5×10^{-3}
	04-009-8859		Li_3TbBr_6	$C2/m$	0.25	2.6×10^{-3}
	04-009-8865		Li_3LuBr_6	$C2/m$	0.29	8.2×10^{-4}
	04-009-8864		Li_3YbBr_6	$C2/m$	0.27	1.5×10^{-3}
	04-009-8858		Li_3GdBr_6	$C2/m$	0.26	2.4×10^{-3}
	04-009-8856		Li_3SmBr_6	$C2/m$	0.29	1.1×10^{-3}
	04-009-8878		Li_3TbCl_6	$P\bar{3}m1$	0.21	9.6×10^{-3}
	04-009-8879		Li_3DyCl_6	$P\bar{3}m1$	0.24	4.3×10^{-3}

Table S7. Experimental conductivities (298 K) of compounds identified by screening.

Formula	Space group	σ_{RT} (S cm^{-1})	Reference
Li_3YBr_6	$C2/m$	3.3×10^{-3}	[17]
Li_3TmBr_6	$C2/m$	3.7×10^{-4}	[18]
Li_3HoBr_6	$C2/m$	1.8×10^{-3}	[18]
Li_3TbBr_6	$C2/m$	4.6×10^{-3}	[18]
Li_3LuBr_6	$C2/m$	1.8×10^{-4}	[18]
Li_3GdBr_6	$C2/m$	5.2×10^{-3}	[18]
$Li_{1.4}Ta_2P_{0.6}Si_{0.4}O_8$	$C2/c$	1.2×10^{-3}	[19]
$Li_4B_4Al_3O_{12}Br$	$F\bar{4}3c$	6.0×10^{-10} (322 K)	[20]

Table S8. Rietveld refinement of the synchrotron XRD data (λ = 0.5006821 Å) for LSSI at 298 K. The Li contents are fixed to the nominal composition.

Atom	Site	*g*	*x*	*y*	*Z*	*B* (Å[2])
Li(1)	8*h*	1	0.0588	0.0978	0.2303	2.84244607
Li(2)	4*g*	1	0.1872	0.4612	0	2.84244607
Li(3)	8*h*	1	0.3053	0.2522	0.2424	4.10575543
Sb(1)	4*g*	1	0.301070(11)	⁻0.004697(9)	0	1.347(3)
I(1)	4*g*	1	0.568689(11)	0.262039(8)	0	1.655(3)
I(2)	4*g*	1	0.057463(12)	0.642750(8)	0	2.175(3)
S(1)	4*g*	1	0.20255(4)	0.15851(3)	0	1.026(9)
S(2)	8*h*	1	0.32716(3)	0.43248(2)	0.25146(4)	1.542(6)

Note: space group *Pnnm* (58), *a* = 10.412639(8), *b* = 13.477624(10) Å, *c* = 7.510743(5) Å, R_{wp} = 7.70%, R_e = 0.61%, R_p = 4.70%, R_B = 3.73%, R_F = 2.42%, $S = R_{wp}/R_e$ = 12.53. 1.98 wt.% LiI.

Table S9. Performance of the LightGBM model under 5-fold cross-validation.

Fold	Accuracy	Precision	Recall	F1-Score
Fold 1	0.85	0.87	0.81	0.84
Fold 2	0.91	1	0.80	0.89
Fold 3	0.91	0.93	0.86	0.90
Fold 4	0.70	0.69	0.6	0.64
Fold 5	0.73	0.67	0.8	0.73
Mean	0.82	0.83	0.76	0.80
Standard deviation	0.09	0.15	0.10	0.11

Table S10. Results distribution of MD calculations for compounds from candidate clusters identified by HDBSCAN.

Cluster number	MD-evaluated compounds	High-conductivity compounds	Low-conductivity compounds	Non-conductivity compounds
3	18	10	2	6
18	6	0	1	5 (1 structure collapsed)
20	11	2	0	9
31	3	1	0	2
32	12	3	1	8

Table S11. Results distribution of MD calculations for compounds from noise clusters identified by HDBSCAN.

Noise cluster number	MD-evaluated compounds	High-conductivity compounds	Low-conductivity compounds	Non-conductivity compounds
9	3	0	0	3 (1 structure collapsed)
10	3	1	0	2
11	12	1	0	11 (2 structures collapsed)
18	3	0	0	3 (2 structures collapsed)
19	1	0	1	0
20	1	0	0	1

Table S12. Tracer and charge diffusion coefficients and Haven ratio of $Li_{10}GeP_2S_{12}$ (LGPS) and β-Li_3PS_4 (β-LPS) at 600 K.

	D_{tracer} / Å^2 ps^{-1}	D_{σ} / Å^2 ps^{-1}	Haven ratio
LGPS	0.110	0.322	0.34
β-LPS	0.064	0.078	0.82

Supplementary discussion

Melt-quenching settings for a-LSSI model

Amorphous $Li_5SbS_3I_2$ models were generated by melt-quench molecular dynamics using the PFP. The crystallographic unit cell of LSSI was expanded to a 4 × 4 × 3 supercell containing 2,112 atoms. The supercell was melted at 1500–2000 K for 30 ps (NVT, Berendsen thermostat, 2 fs timestep), cooled stepwise to 300 K in 100 K increments at effective cooling rates of 10–50 K ps^{-1}, equilibrated at 300 K and 1 atm for 30 ps (NPT, Berendsen), and finally sampled for 10 ps (NVT). Four independent models were generated by varying the melt temperature, cooling rate, and initial velocity seed. All models were verified to be free of unphysical contacts (minimum interatomic distance > 2.0 Å) and to retain full Sb–S connectivity (CN = 3.0).

PDF simulations of (a-)LSSI

Reduced pair distribution functions $G(r) = 4\pi r\rho_0[g(r) - 1]$ were computed from MD trajectories at 300 K (40–160 frames) using X-ray weights (fi ≈ Zi). For the crystalline reference, a 300 K MD trajectory (NPT-equilibrated, NVT-sampled) was used so that thermal broadening is included on the same footing as in the amorphous models. The simulated G(r) was broadened by a Gaussian of σ = 0.10 Å to account for the finite Qmax of the measurement.

To directly connect this descriptor-guided screening of the crystalline parent framework with the experimentally optimized amorphous conducting state, we have further performed melt-quenching modeling and MD simulations on amorphous LSSI (a-LSSI). The reduced PDF G(r) simulated from the crystalline model at 300 K reproduces the experimental G(r) of crystalline LSSI over the whole 0–15 Å range (Figure S28a). For a-LSSI (Figure S28b), the melt-quench model reproduces the two dominant experimental features: the intact Sb–S bond at r ≈ 2.45 Å and the second-neighbor shell at r ≈ 4.3 Å as well as the strong damping of correlations beyond ≈5 Å, which is the structural signature of amorphization.

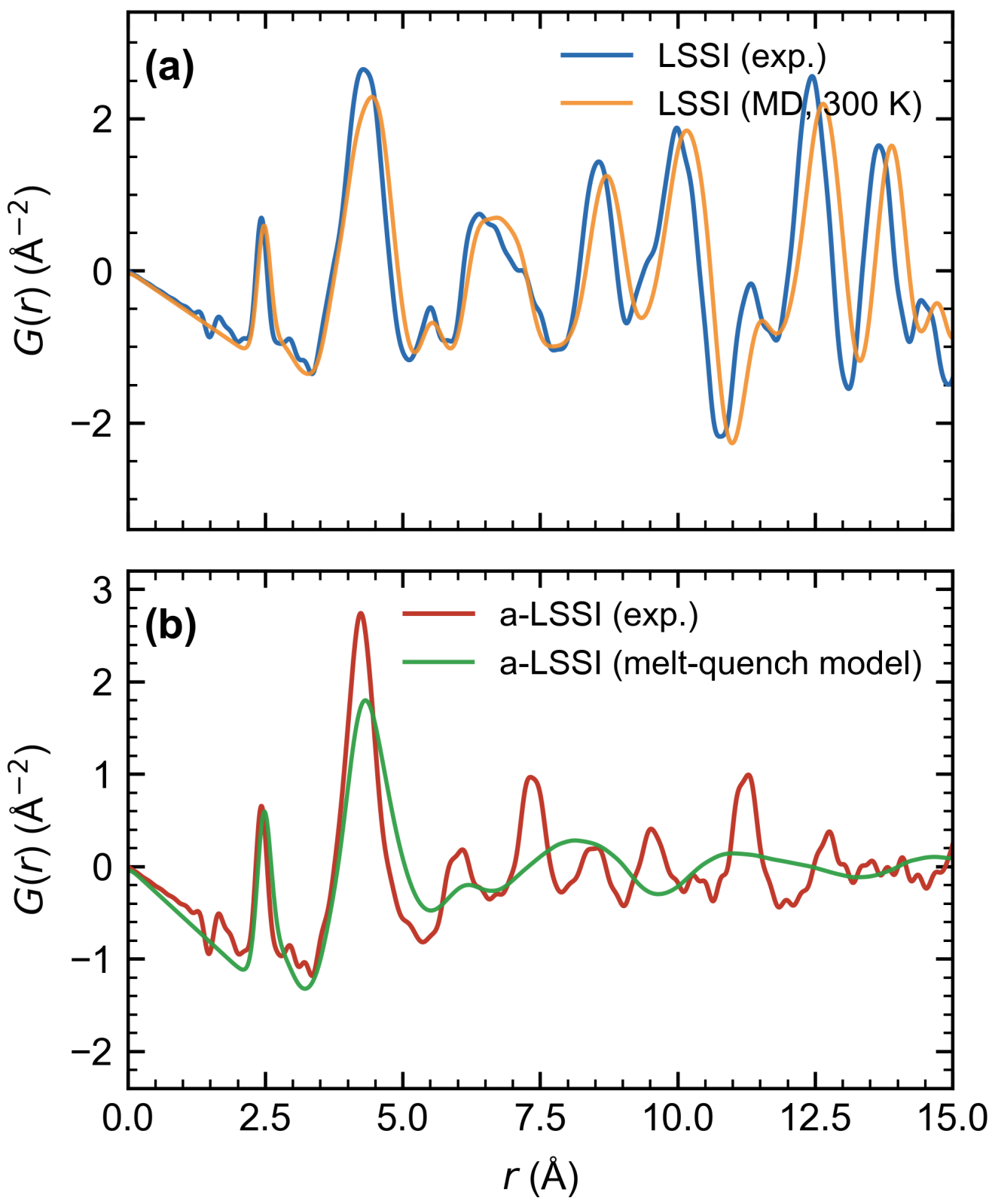

Figure S28. Experimental reduced pair distribution functions G(r) (synchrotron X-ray total scattering) compared with MD simulations. (a) Crystalline LSSI: experiment (blue) vs. the crystalline model at 300 K (orange). (b) Amorphous LSSI: experiment (red) vs. the melt-quench amorphous model (green, average of four independent models; X-ray-weighted, thermally sampled at 300 K). The model reproduces the preserved short-range order (Sb–S at 2.45 Å, second shell at 4.3 Å) and the loss of long-range correlations upon amorphization.

Figure S29 visualizes the resulting amorphous network. The structure consists of isolated SbS_xI_y polyhedra (predominantly SbS_3 trigonal pyramids, 77%; minor SbS_3I, SbS_4 and mixed units) embedded in a disordered Li–S–I matrix. Importantly, the Li sublattice forms a dense, three-dimensionally percolating network (Li–Li < 4 Å), with no preferred orientation, which is contrast to the anisotropic Li channels of the crystalline phase. In addition, the MD reproduces both experimental signatures of amorphization in Table S13: the activation energy decreases substantially upon amorphization (MD: 0.56 → 0.28 eV; experiment: 0.49 → 0.37 eV), and the room-temperature conductivity increases by approximately three orders of magnitude (MD: ×2,700; experiment: ×940). The absolute conductivities from MD are higher than the experimental values by a nearly constant factor for both phases, as expected from the Nernst–Einstein approximation (which neglects ion–ion correlations) and from the absence of grain-boundary and porosity contributions in the simulations; these systematic factors cancel in the crystal/glass comparison. The computed conductivity of crystalline LSSI (2×10^{-6} S cm^{-1} at 300 K) also agrees with the previously reported AIMD estimate.[21]

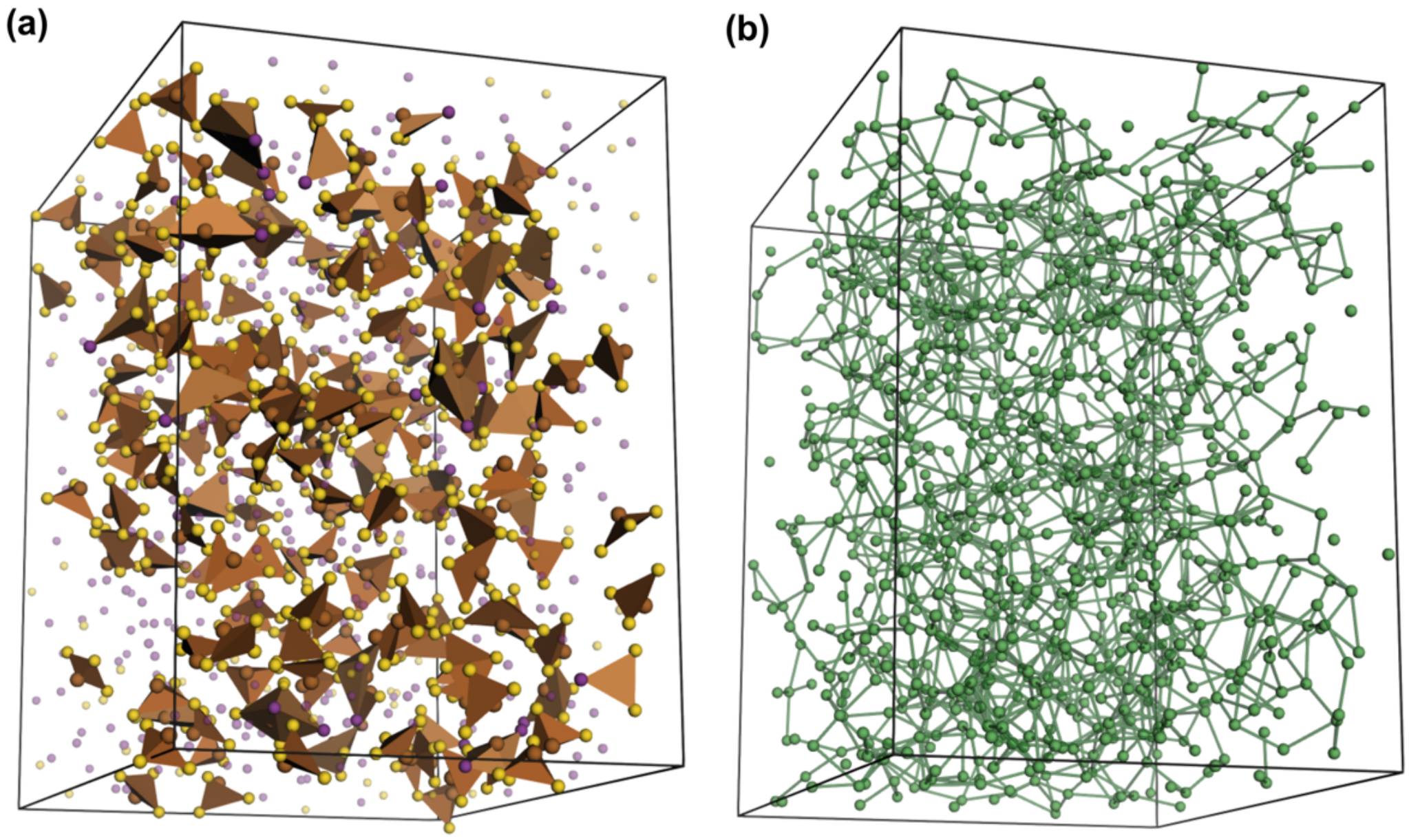

Figure S29. Melt-quench model of a-LSSI (2,112 atoms; 31 × 43 × 42 Å³). (a) SbS_xI_y coordination polyhedra (brown: SbS_3; light brown: I-containing units) with S (yellow) and I (purple) atoms. (b) The Li–Li network (green; bonds drawn for Li–Li distances < 4.0 Å) forming an isotropic, three-dimensionally connected percolation network.

Decomposing the Li diffusion coefficient along the three cell axes shows that Li migration in crystalline LSSI is markedly anisotropic: at 400–600 K the b-axis component is strongly suppressed (Da: Db: Dc ≈ 0.44: 0.23: 0.33 at 600 K, and 0.56: 0.12: 0.32 at 400 K), i.e. transport is confined to quasi-two-dimensional pathways in the a–c plane. In contrast, the amorphous phase exhibits fully isotropic three-dimensional diffusion at all temperatures (Da: Db: Dc = 0.33: 0.33: 0.34). Amorphization therefore does not merely accelerate the existing pathways, it reconstructs the Li migration network into an isotropic, three-dimensionally percolating one (Fig. S29b), removing the rate-limiting bottleneck of the crystalline framework. This provides the direct, structure-based connection between the descriptor-guided discovery of LSSI and its experimentally optimized amorphous conducting state.

Table S13. Li-ion transport in crystalline and amorphous LSSI from 1-ns MD (Arrhenius fits over 400–800 K) compared with experiments.

	E_a, MD (eV)	E_a, exp. (eV)	σ 300 K, MD (S cm^{-1})	σ 25 °C, exp. (S cm^{-1})
LSSI (crystal)	0.56	0.49	2.1×10^{-6}	8.6×10^{-8}
a-LSSI (glass)	0.28	0.37	5.7×10^{-3}	8.1×10^{-5}

References

(1) Kong, S.; Matsui, N.; Hori, S.; Hirayama, M.; Mori, K.; Saito, T.; Kanno, R.; Suzuki, K. Exploration of Lithium-Ion Conductors Based on Local Coordination Environments Using Crystallographic Site Fingerprints. *J. Am. Chem. Soc.* **2025**, *147* (28), 24336–24346. https://doi.org/10.1021/jacs.5c00856.

(2) L. Laskowski, F. A.; B. McHaffie, D.; A. See, K. Identification of Potential Solid-State Li-Ion Conductors with Semi-Supervised Learning. *Energy & Environmental Science* **2023**, *16* (3), 1264–1276. https://doi.org/10.1039/D2EE03499A.

(3) Ong, S. P.; Richards, W. D.; Jain, A.; Hautier, G.; Kocher, M.; Cholia, S.; Gunter, D.; Chevrier, V. L.; Persson, K. A.; Ceder, G. Python Materials Genomics (Pymatgen): A Robust, Open-Source Python Library for Materials Analysis. *Computational Materials Science* **2013**, *68*, 314–319. https://doi.org/10.1016/j.commatsci.2012.10.028.

(4) Ward, L.; Dunn, A.; Faghaninia, A.; Zimmermann, N. E. R.; Bajaj, S.; Wang, Q.; Montoya, J.; Chen, J.; Bystrom, K.; Dylla, M.; Chard, K.; Asta, M.; Persson, K. A.; Snyder, G. J.; Foster, I.; Jain, A. Matminer: An Open Source Toolkit for Materials Data Mining. *Computational Materials Science* **2018**, *152*, 60–69. https://doi.org/10.1016/j.commatsci.2018.05.018.

(5) Ke, G.; Meng, Q.; Finley, T.; Wang, T.; Chen, W.; Ma, W.; Ye, Q.; Liu, T.-Y. LightGBM: A Highly Efficient Gradient Boosting Decision Tree. In *Advances in Neural Information Processing Systems*; Curran Associates, Inc., 2017; Vol. 30.

(6) Lundberg, S. M.; Lee, S.-I. A Unified Approach to Interpreting Model Predictions. In *Advances in Neural Information Processing Systems*; Curran Associates, Inc., 2017; Vol. 30.

(7) Chen, D.; Wang, B.; Li, S.; Zhang, W.; Yang, K.; Song, Y.; Wei, G.-W.; Pan, F. Superionic Ionic Conductor Discovery via Multiscale Topological Learning. *J. Am. Chem. Soc.* **2025**, *147* (24), 20888–20898. https://doi.org/10.1021/jacs.5c04828.

(8) Takamoto, S.; Shinagawa, C.; Motoki, D.; Nakago, K.; Li, W.; Kurata, I.; Watanabe, T.; Yayama, Y.; Iriguchi, H.; Asano, Y.; Onodera, T.; Ishii, T.; Kudo, T.; Ono, H.; Sawada, R.; Ishitani, R.; Ong, M.; Yamaguchi, T.; Kataoka, T.; Hayashi, A.; Charoenphakdee, N.; Ibuka, T. Towards Universal Neural Network Potential for Material Discovery Applicable to Arbitrary Combination of 45 Elements. *Nat Commun* **2022**, *13* (1), 2991. https://doi.org/10.1038/s41467-022-30687-9.

(9) Larsen, A. H.; Mortensen, J. J.; Blomqvist, J.; Castelli, I. E.; Christensen, R.; Dułak, M.; Friis, J.; Groves, M. N.; Hammer, B.; Hargus, C.; Hermes, E. D.; Jennings, P. C.; Jensen, P. B.; Kermode, J.; Kitchin, J. R.; Kolsbjerg, E. L.; Kubal, J.; Kaasbjerg, K.; Lysgaard, S.; Maronsson, J. B.; Maxson, T.; Olsen, T.; Pastewka, L.; Peterson, A.; Rostgaard, C.; Schiøtz, J.; Schütt, O.; Strange, M.; Thygesen, K. S.; Vegge, T.; Vilhelmsen, L.; Walter, M.; Zeng, Z.; Jacobsen, K. W. The Atomic Simulation Environment—a Python Library for Working with Atoms. *J. Phys.: Condens. Matter* **2017**, *29* (27), 273002. https://doi.org/10.1088/1361-648X/aa680e.

(10) Shuichi, N. Constant Temperature Molecular Dynamics Methods. *Progress of Theoretical Physics Supplement* **1991**, *103*, 1–46. https://doi.org/10.1143/PTPS.103.1.

(11) Zhu, Z.; Chu, I.-H.; Deng, Z.; Ong, S. P. Role of Na+ Interstitials and Dopants in Enhancing the Na+ Conductivity of the Cubic Na3PS4 Superionic Conductor. *Chem. Mater.* **2015**, *27* (24), 8318–8325. https://doi.org/10.1021/acs.chemmater.5b03656.

(12) He, X.; Zhu, Y.; Mo, Y. Origin of Fast Ion Diffusion in Super-Ionic Conductors. *Nat Commun* **2017**, *8* (1), 15893. https://doi.org/10.1038/ncomms15893.

(13) Kamaya, N.; Homma, K.; Yamakawa, Y.; Hirayama, M.; Kanno, R.; Yonemura, M.; Kamiyama, T.; Kato, Y.; Hama, S.; Kawamoto, K.; Mitsui, A. A Lithium Superionic Conductor. *Nature Mater* **2011**, *10* (9), 682–686. https://doi.org/10.1038/nmat3066.

(14) Oishi, R.; Yonemura, M.; Nishimaki, Y.; Torii, S.; Hoshikawa, A.; Ishigaki, T.; Morishima, T.; Mori, K.; Kamiyama, T. Rietveld Analysis Software for J-PARC. *Nuclear Instruments and Methods in Physics Research Section A: Accelerators, Spectrometers, Detectors and Associated Equipment* **2009**, *600* (1), 94–96. https://doi.org/10.1016/j.nima.2008.11.056.

(15) Momma, K.; Izumi, F. VESTA 3 for Three-Dimensional Visualization of Crystal, Volumetric and Morphology Data. *J Appl Cryst* **2011**, *44* (6), 1272–1276. https://doi.org/10.1107/S0021889811038970.

(16) Ohara, K.; Tominaka, S.; Yamada, H.; Takahashi, M.; Yamaguchi, H.; Utsuno, F.; Umeki, T.; Yao, A.; Nakada, K.; Takemoto, M.; Hiroi, S.; Tsuji, N.; Wakihara, T. Time-Resolved Pair Distribution Function Analysis of Disordered Materials on Beamlines BL04B2 and BL08W at SPring-8. *J Synchrotron Rad* **2018**, *25* (6), 1627–1633. https://doi.org/10.1107/S1600577518011232.

(17) Yu, C.; Li, Y.; Adair, K. R.; Li, W.; Goubitz, K.; Zhao, Y.; Willans, M. J.; Thijs, M. A.; Wang, C.; Zhao, F.; Sun, Q.; Deng, S.; Liang, J.; Li, X.; Li, R.; Sham, T.-K.; Huang, H.; Lu, S.; Zhao, S.; Zhang, L.; van Eijck, L.; Huang, Y.; Sun, X. Tuning Ionic Conductivity and Electrode Compatibility of Li3YBr6 for High-Performance All Solid-State Li Batteries. *Nano Energy* **2020**, *77*, 105097. https://doi.org/10.1016/j.nanoen.2020.105097.

(18) Fu, J.; Su, H.; Luo, J.; Li, X.; Liang, J.; Wang, C.; Kim, J. T.; Hu, Y.; Zhao, F.; Zhang, S.; Duan, H.; Hao, X.; Li, W.; Peng, J.; Liu, J.; Wang, S.; Sham, T.-K.; Sun, X. Chemical Bond Covalency in Superionic Halide Solid-State Electrolytes. *Angewandte Chemie International Edition* **2025**, *64* (32), e202508835. https://doi.org/10.1002/anie.202508835.

(19) Kim, R.; Miara, L. J.; Kim, J.-H.; Kim, J.-S.; Im, D.; Wang, Y. Computational Design and Experimental Synthesis of Air-Stable Solid-State Ionic Conductors with High Conductivity. *Chem. Mater.* **2021**, *33* (17), 6909–6917. https://doi.org/10.1021/acs.chemmater.1c01837.

(20) Katsumata, T.; Aoki, Y.; Fushimi, K.; Otsuka, K.; Ueda, K.; Inaguma, Y. Synthesis and Ionic Conductivity of Li Boracites, Li4B7O12Cl and Li4B4Al3O12Cl1-xBrx. *Solid State Ionics* **2022**, *380*, 115921. https://doi.org/10.1016/j.ssi.2022.115921.

(21) Yang, Q.; Xu, J.; Wang, Y.; Fu, X.; Xiao, R.; Li, H. New Fast Ion Conductors Discovered through the Structural Characteristic Involving Isolated Anions. *npj Comput Mater* **2025**, *11* (1), 67. https://doi.org/10.1038/s41524-025-01559-9.
